# Supplementary material for: Widespread 2013-2020 decreases and reduction challenges of organic aerosol in China
Source: Nat Commun. 2024 May 25;15:4465. doi: 10.1038/s41467-024-48902-0 (PMC11127919; doi:10.1038/s41467-024-48902-0)
Supplement: Supplementary file 1 — Supplementary Information [file 41467_2024_48902_MOESM1_ESM.pdf]

Supplementary Information for

Qi Chen *et al.*

*Corresponding author: Qi Chen

Email: qichenpku@pku.edu.cn

This PDF file includes:

Note S1, Note S2, Note S3
Figures S1 to S23
Tables S1 to S9
Supplementary References

Supplementary Notes

Note S1. Description of Model Simulations

Model configurations. GEOS-Chem is a widely used atmospheric chemical transport model. In this study, we used the GEOS-Chem model version 13.3.1 (DOI: 10.5281/zenodo.5703364) driven by the MERRA2 reanalysis meteorological fields from NASA Global Modeling and Assimilation Office. The nested-grid simulations were run for China (15°-55° N, 70-140° E) with a horizontal resolution of 0.5° × 0.625° and a vertical resolution of 47 levels extending to 0.01 hPa. The boundary conditions were obtained from global simulations at 2° × 2.5° horizontal resolution. Both of the nested and global simulations were performed with a spin-up period of one month.

The model simulates the ozone-NO_x-hydrocarbon-aerosol-halogen chemistry in the troposphere coupled with the ISORROPIA-II thermodynamic module for sulfate-nitrate-ammonium aerosols and the revised complex SOA scheme for organic aerosol (OA) [1-3]. Advection is calculated by a flux-form semi-Lagrangian scheme [4]. Boundary layer mixing is represented by a non-local scheme [5]. Convective transport uses archived convective mass fluxes from MERRA2. The dry deposition is calculated by a standard resistance-in-series model with increased dry deposition velocities at low temperatures [6-8]. The wet deposition of gases and particles considers the scavenging through wet convective updrafts and large-scale precipitation with updates for major inorganic aerosols and their precursors [9,10].

The Community Emissions Data System (CEDS) provides global anthropogenic emissions for countries other than China and ship emissions [11]. The biogenic and open biomass burning emissions are calculated from the Model of Emissions of Gases and Aerosols from Nature (MEGAN v2.1) and the Global Fire Emission Database with small fire (GFED4s) [12,13]. Natural NO_x emissions from lightning and soil are also included [14,15]. Anthropogenic emissions from five sectors such as solvent use, industry, power, residential, and transportation in China are provided by Multi-resolution Emission Inventory for China (MEIC), among which the emissions from industry, residential, and transportation are further divided by fuel types such as industrial coal burning, industrial other sources, residential biofuel burning, residential coal burning, residential other sources, diesel engines, and gasoline engines. Agriculture ammonia emissions are adopted from Chen, et al. [16] for 2013 to 2016 and scaled for the years after 2017 according to the trend from MEIC agriculture ammonia emissions.

NMVOC emissions and VOC-type OA precursors. Compared to the original version of MEIC [17,18], residential biofuel and coal use are updated on the basis of nationwide on-site surveys [19]. Therefore, the non-methane VOC (NMVOC) emissions herein are slightly different from the original version [18]. From 2013 to 2017, control measures such as newly-installed leak detection and repair systems in petrochemical industry, the replacement of residential coal or biofuel burning with electrical or gas stoves, the upgrade of vehicle emission standards and so on together led to about 6% reduction of the NMVOC emissions. This reduction was offset by the increased emissions from expanded activities in solvent use and industry. The net change is an increase of the NMVOC emissions from 26.6 to 28.2 Tg y^{-1} in China during the period of 2013 to 2016. Then the annual NMVOC emissions stayed high at 27.7 Tg y^{-1} from 2017 to 2018 and were reduced by 0.7 Tg in 2019 as the vehicle emission standards were tightened to China V and the replacement of residential stoves continued. In 2020, the annual NMVOC emissions decreased

by 1.4 Tg as a result of the significant shrink of economic activities after the COVID-19 outbreak. Among NMVOC, aromatic compounds, oxygenated VOCs (OVOCs) such as glyoxal (GLY) and methylglyoxal (MGLY), isoprene, and terpenes are common SOA precursors which account for about 34-37% of the NMVOC emissions in China during 2013-2020 [20]. The emissions of aromatic compounds show an increase from 8.7 to 10.0 Tg y^{-1} during the period of 2013 to 2018, mainly contributed by the expansion of solvent use [18]. By contrast, the primary emissions of GLY+MGLY (i.e., 0.2 Tg y^{-1}) show a continuous annual reduction of 0.01 Tg from 2013 to 2020 as a result of the replacement of residential fuels. The anthropogenic emissions of isoprene and monoterpenes have little change, and their emissions (i.e., 0.1 Tg y^{-1}) are quite small compared to their natural emissions.

IVOC emissions and volatility distributions. The emissions of IVOC and S/LVOC are estimated following the methods introduced in our previous study [3]. IVOC emissions from industry, solvent use, power, transportation, residential, and open biomass burning are scaled from their NMVOC emissions and the ratios of emission factors between IVOC and NMVOC (EF_{IVOC}/EF_{NMVOC}) according to the better correlation of IVOC with NMVOC than with individual IVOC species (e.g., naphthalene) or POA [21,22]. The values of EF_{IVOC}/EF_{NMVOC} for each sector are listed in Table S4. Specifically, the EF_{IVOC}/EF_{NMVOC} for residential biofuel burning is averaged from the domestic combustion results of corn straw, rice straw, and wheat straw in China and 16 types of fuel woods and 3 types of crop residues in India [23,24]. The EF_{IVOC}/EF_{NMVOC} from combustion of bituminous coal is used to represent residential coal burning in China [25,26]. The EF_{IVOC}/EF_{NMVOC} for residential other burning is adopted from the combustion of municipal solid waste because waste treatment contributes predominantly to other fuel burning in the residential sector [18,24]. The EF_{IVOC}/EF_{NMVOC} from combustion of anthracite coal is used for industrial coal burning and power [25,26]. The EF_{IVOC}/EF_{NMVOC} for industrial other burning is adopted from Qi, et al. [27]. The EF_{IVOC}/EF_{NMVOC} for diesel engines and gasoline engines are averaged from several experiments under different vehicle types and operating conditions, respectively [28-30]. The EF_{IVOC}/EF_{NMVOC} for solvent use is averaged from the estimations of industry paint, pesticide use, and dry clean from Seltzer, et al. [31] and the measurements of architecture coating conducted by Tanzer-Gruener, et al. [32]. The EF_{IVOC}/EF_{NMVOC} for open biomass burning is taken from the average of biomass burning experiments from FIREX and FLAME4 [33,34]. Only for ship emissions, the ratio of emission factors between IVOC and POA (EF_{IVOC}/EF_{POA}) is used because the correlation of IVOC with POA is much better than with NMVOC [35]. The value of EF_{IVOC}/EF_{POA} is averaged from the ratios reported for low-sulfur fuel and high-sulfur fuel vessels [35]. IVOC emissions are further distributed to three volatility bins with saturation concentrations (C^*) of 10^6, 10^5, and $10^{\leq 4}$ according to the averages of corresponding laboratory results described above (Table S4).

S/LVOC emissions and volatility distributions. Emissions of S/LVOC are estimated from the primary OC emissions provided by MEIC for industry, transportation, power, and residential sectors, by GFED4s for open biomass burning, and by CEDS for shipping with a filter-artifact scaling factor of 1.0. An organic-matter-to-OC ratio (OM:OC) of 1.4 is used for converting readily-condensable S/LVOC to POA mass [36]. S/LVOC emissions are divided into five volatility bins with C^* from 10^{-2} to 10^2 μg m^{-3}. The volatility distributions of S/LVOC for each sector are the averaged distributions reported from laboratory combustion experiments except that the distribution from May, et al. [37] is used for open biomass burning (Table S5).

Uncertainty of NMVOC, IVOC, S/LVOC emissions. The uncertainties for VOC and SVOC (i.e., POC in MEIC) emissions are ±68% and -44%-92%, respectively, for 95% confidence intervals (CI) [38]. The uncertainty for IVOC emissions is estimated by the Monte Carlo method that propagates the uncertainties of VOC emission and the values of EF_{IVOC}/EF_{NMVOC} to total IVOC emissions. The probabilistic distributions of EF_{IVOC}/EF_{NMVOC} for individual source sectors are confirmed by Kolmogorov–Smirnov test with corresponding experimental results. The Monte Carlo simulations are conducted 10000 times and show a 95% CI of -68% to 92% for IVOC emissions. The uncertainties for individual source sectors can be greater depending on the availability and the inventory adoption of the emission measurements [26,38].

Model modifications. We have made several updates on the formation of inorganic aerosol, HONO sources, and the uptake coefficient of HO_2 (γ_{HO2}) to the default model according to our previous work [3]. The heterogeneous oxidation of SO_2 on wet aerosols is added for sulfate formation in addition to traditional SO_2 oxidation in the gas phase and cloud droplets [39-42]. The uptake coefficient of SO_2 (γ_{SO2}) is set to be 10^{-6}. The parameterization of HNO_3 formation mainly follows Holmes, et al. [43] except that the uptake coefficient of NO_2 on wet aerosols ($\gamma_{NO2\text{-}a}$) for black carbon (BC) is set to 10^{-5} instead of 10^{-4} in this study [44]. Primary HONO emissions from traffic, soil, and open biomass burning are implemented by scaling from corresponding NO_x emission for the former two and adding combustion-type-dependent HONO emission factors to GFED4s for the latter one [45-47]. Besides the default HONO formation from homogeneous gas phase reaction and heterogeneous oxidation on wet aerosols, the heterogeneous oxidation of NO_2 on the ground is added to the surface layer in the model that depends on the mean molecular speed of NO_2, the ground-surface-to-volume ratio (S_g/V), and the uptake coefficient of NO_2 on the ground ($\gamma_{NO2\text{-}g}$) [48]. The S_g/V is fixed to 0.1 m^{-1} for urban areas and depends on the leaf area index and boundary layer height in non-urban areas [49,50]. The value of $\gamma_{NO2\text{-}g}$ linearly increases with light intensity and relative humidity (RH) [44,51]. Moreover, the photolysis of nitrate is implemented by using a ratio of 50 for the photolysis rate of nitrate versus HNO3 (J_{scale}) [52]. In addition, the value of γ_{HO2} is decreased to 0.08 from 0.2 based on the observations in NCP [53]. All the model modifications apart from the default version are listed in Table S6.

SOA simulations and updates. In the Complex SOA scheme, POA is treated as S/LVOC that is usually emitted as particles without undergoing oxidation near sources and is able to repartition into the gas phase after dilution [54]. The gas-phase S/LVOC is oxidized by OH radicals at a rate constant of 2×10^{-11} cm^3 $molec^{-1}$ s^{-1} in the model. The oxidation products have 100-fold lower C^* than their precursors and can partition to the particle phase to form SOA. SOA is also formed from the oxidation of lumped biogenic VOC (BVOC), aromatic VOC (AVOC), and IVOC [3,55]. The SOA formed from AVOC and IVOC through photooxidation and those from the oxidation of terpenes by OH and O_3 are parameterized by the VBS approach with NO_x-dependent SOA yields as listed in Table S7. The SOA yields for AVOC and IVOC are corrected for vapor wall loss by multiplying 1.9/1.2 and 1.2/1.2 under low/high-NO_x conditions, respectively [56]. The oxidation of isoprene by OH under the low-NO_x condition and the oxidations of isoprene and monoterpene by NO_3 are replaced by the explicit chemical treatments of aqueous-phase uptake of isoprene oxidation products mainly isoprene epoxydiols (IEPOX) and the formation of BVOC-derived organic nitrate, respectively [57-59]. The uptake coefficients of isoprene oxidation products depend on their effective Henry's Law constant and aqueous-phase reaction rate constant [57]. Besides, the scheme includes the heterogeneous uptakes of glyoxal and methylglyoxal in clouds and aerosols to form SOA with fixed uptake coefficients [57]. Salting-in and salting-out effects of IEPOX,

glyoxal, and methylglyoxal are not considered in this study, which is however expected to have negligible impacts on the modeled SOA concentrations because of the small contribution of aqueous SOA to the total mass loadings [60]. The OM:OC ratio for SOA formed from gas-phase oxidations is 2.1 [61]. For the SOA that formed from aqueous-phase uptake, the OM-to-OC ratios depends on the carbon numbers of precursors. Both POA and SOA can be removed by dry deposition and wet deposition. The scavenging efficiencies of POA and SOA in wet deposition is set to be 50% and 80%, respectively [3].

Model scenarios. As listed in Table S8, we performed a base simulation (Base) from 2013 to 2020 and 18 sensitive simulations to investigate the impact of variations in emissions and meteorology on OA concentrations in China. For the analysis of driven factors during the two action-plan periods (Fig. 3*A, C*), the four sensitive simulations (CTL_ALLEMIS, CTL_OAP, CTL_OTR, and CTL_MET) were run for the years 2017 and 2020. Emissions of all pollutants, OA precursors, and non-OA-precursor pollutants as well as meteorological fields are fixed to the year of 2013 or 2017 under CTL_ALLEMIS, CTL_OAP, CTL_OTR, and CTL_MET senarios, respectively. The concentration variations caused by corresponding factors are calculated as the differences in POA or SOA concentrations between the Base and sensitive simulations. For example, the impact of meteorological variations is calculated by $Base_i$ - CTL_MET_i for which i is the simulation year. The impact of emission controls on non-OA-precursor pollutants is calculated by $Base_i$ - CTL_OTR_i. The impact of emission controls on OA precursors are calculated by $Base_i$ - CTL_OAP_i and $CTL\_ALLEMIS_i$ - CTL_OTR_i. The two equations differ slightly by the base-year pollution level. We include the former in Fig. 3. In fact, the similar results for the two calculations suggest negligible nonlinear effects of the base-year pollution level for the period of study. Similarly, CTL_OTR_woSO_2 and CTL_OTR _woNO_x were run to quantify the contribution of emission controls of SO_2 and NO_x emissions to the concentration variations (Fig. S15), which are calculated by $CTL\_OTR\_woSO_{2\,i}$ - CTL_OTR_i and $CTL\_OTR\ \_woNO_{x\,i}$ - CTL_OTR_i, respectively. The impact of other pollutants is calculated by subtracting the contributions of SO_2 and NO_x emission changes from the contributions of emission changes in all non-OA-precursor pollutants. Furthermore, the simulations of Fix_ALLEMIS and Fix_MET were conducted from 2014 to 2020 with fixed 2013-emission (all sources) and 2013-meteorology, respectively. The yearly percent changes of concentrations relative to 2013 due to meteorology and emission (Fig. 3*B, D*) are calculated by $Fix\_ALLEMIS_i$ / $Base_{2013}$-1 and Fix_MET_i / $Base_{2013}$-1, respectively. Finally, the simulations of Half_ and Zero_ series were conducted to investigate the impacts of controlling anthropogenic emissions of OA precursors in residential, solvent use, and industry sectors. The concentration changes relative to the 2019 level (Fig. 4) are calculated by the differences between $Base_{2019}$ and the corresponding cases.

Random forest model and SHAP analysis. A random forest (RF) model is used to investigate the meteorology impacts on POA and SOA concentrations [62]. The RF model is an ensemble of decision trees that can estimate feature importance by calculating the decrease in impurity. The feature importance can reveal key factors for the meteorology impacts on POA and SOA as well as their differences. Meteorology-driven concentration changes of POA and SOA compared to the year 2013 are set to the target variable to the RF model, which are calculated through the differences between the concentrations in Fix_ALLEMIS from 2014 to 2020 and $Base_{2013}$ as shown in Table S8. For each year between 2014 to 2020, we developed RF model simulations to reproduce the annual mean meteorology-driven concentration changes.

The annual changes in meteorology factors compared to the year 2013 are used as input features. The optimal hyperparameters (n_estimators=100, max_features=1/3 of the number of input features, bootstrap= "True") and input features are used after tuning. The input features include meteorological factors such as air temperature at 2 m (T), the east-west and north-south wind components at 10 m (U and V), surface shortwave radiation (SWR), and precipitation at ground, the model first layer parameters such as pressure ($Pressure_1$), RH_1, specific humidity (SH_1), cloud fraction ($CLD\text{-}Frac_1$), and cloud optical thickness ($CLD\text{-}OT_1$), and other factors related to vertical mixing and dry deposition such as friction velocity (U^*), planetary boundary layer height (PBLH), and tropopause pressure ($Pressure_{tp}$). Surface concentrations (CONC) of POA or SOA in 2013 can also be included in the model input, which can slightly improve the RF *r* values (Fig. S12). The RF models are trained by 70% of the dataset and validated by the rest 30% data. As shown in Fig. S12, the RF model can reasonably learn the nonlinear relationships between interannual variability of meteorology and meteorology-driven concentration changes with the *r* values of 0.94 and 0.96 for POA and SOA, respectively.

The feature importance is averaged from the yearly results. For the RF model with concentrations as input features, the mean feature importance values with standard deviations are shown in Fig. 3*F*. The meteorology-driven concentration changes of POA and SOA are both highly dependent on their concentrations in 2013 while showing different responses to individual meteorology factors. POA is more sensitive to factors relative to pressure and temperature that influence transport and vertical mixing processes. Cloud-related factors, specific humidity, and friction velocity show higher importance on SOA. The first two may suggest SOA is sensitive to factors that affect photochemistry processes. The latter may reveal the influence of dry deposition on SOA precursors. The mean feature importance from the RF model with or without using concentrations as input features show similar results, although the values of the feature importance are different (Fig. S13*A*).

Further quantification of meteorological influences for POA and SOA is achieved by using SHapley Additive exPlanation (SHAP) regression values [63]. SHAP is a popular method for explaining the predictions of tree-based machine learning models. It can assign an importance value to each feature by considering all possible combinations of features and measuring their impact on the prediction, accounting for the interactions between features. SHAP values can be positive or negative, presenting that the corresponding feature reduces or raises the prediction from the base value. Therefore, the absolute SHAP (|SHAP|) value can represent the influence of the corresponding feature on the model predictions.

The mean |SHAP| values of meteorological inputs to the meteorology-driven interannual variations of POA and SOA resolved by random forest model analysis are shown in Fig. S13*B*. The rankings for the mean absolute SHAP values of POA and SOA are generally consistent with the mean feature importance RF models. The comparison of the mean |SHAP| values between POA and SOA shows that SOA is more sensitive to cloud-related factors, specific humidity, and friction velocity, similar to the results from feature importance.

Note S2. Description of Ambient Observations

Long-term organic carbon measurements. The annual-mean concentrations of particulate OC are obtained from continuous measurements for $PM_{2.5}$ chemical composition at 40 monitoring sites and 9 research sites in NCP and YRD regions. The NCP monitoring network consists of 34 sites in 28 cities (Fig. 1 and Fig. S5). Annual-mean OC concentrations from both filter-based measurements and online measurements are provided by the China National Environmental Monitoring Centre (CNEMC) for the 34 sites. The data coverage of the NCP network is 2017-2021 for filter-based measurements and 2019-2021 for online measurements. For YRD, we included the long-term on-line measurement data from six provincial monitoring sites. Three sites are located in Zhejiang province and the data are provided by Zhejiang Ecological and Environmental Monitoring Center. One is located at Shanghai and the other two are located in Jiangsu province, for which the data were taken from literature [64-66]. The data with annual data coverage lower than 75% were only used for model-observation comparisons (Fig. S5*A,B*). Moreover, we collected the online and offline measurement data of annual-mean OC concentrations at 5 research sites in NCP [67-70] and 4 research sites in YRD from the literature [71-74] which are included in Fig. S5*C,D*. Offline filter-based measurements analyze daily Quartz filter samples or one sample per three days, and the analysis follows the IMPROVE_A heating protocol. A correction factor of 0.61 was applied to correct the sampling bias from semivolatile organic vapors [75]. Online measurements were conducted by using semi-continuous OC-EC field analyzers (Sunset Model 4, Talroad TR20N9, Focused Photonics OCEC-100) with denuders in front of the sampling line.

Derived primary and secondary organic aerosol concentrations. We collect a large data set for surface concentrations of organic aerosol (OA) and its components from individual campaigns from literature (Table S1, Figs. 1 and S5). This data set contains 161 surface measurements (126 measurements after removing overlapped ones) from 2013 to 2020 that cover the main regions in China including NCP, YRD, Pearl River Delta (PRD), and Northwest China (NW), and some other regions (OTR) [76-184]. In these studies, OA was measured by Aerodyne aerosol mass spectrometers or aerosol chemical speciation monitors, and the mass spectra of OA were analyzed by the positive matrix factorization (PMF) with the PMF2 or ME2 solvers to resolve the OA factors that may represent various sources [185]. We summed the campaign-average concentrations of hydrocarbon-like (HOA), cooking-related (COA), biomass-burning-related (BBOA), and coal-combustion-related (CCOA) OA factors to represent primary organic aerosol (POA). In some studies, some of those factors are resolved as fossil fuel OA, and some of these primary factors might be named differently. We summed the campaign-average concentrations of the oxygenated OA factors (OOAs) to represent secondary organic aerosol (SOA).

Organic precursor measurements. We collect a large data set for nationwide surface concentrations of intermediate volatility organic compounds (IVOC) from individual campaigns from literature (Fig. S19*A*). This data set consists of 7 measurements from literature [186-190]. The sampling sites are located in Shanghai (YRD) and Guangzhou (PRD). IVOC was sampled by sorption tubes and analyzed by thermal desorption gas chromatography/mass spectrometry (TD-GC-MS). All measurements were conducted during 2016 to 2019.

Derived volatility distributions of IVOC and POA. The reported IVOC volatility distributions typically have 11 volatility bins according to their retention times in the GC analysis [191]. We

summed up the B12–B14, B15–B16, and B17-B22 to represent the three volatility bins with C^* of 10^6, 10^5, and $10^{\leq 4}$ μg m^{-3}, respectively (Fig. S20). The modeled surface IVOC concentrations represent only the gas phase. Although the measurements usually include both of gas- and particle-phase IVOC, gas-phase IVOC contributes predominantly to the total mass [190]. The volatility distribution of POA was estimated from the data obtained from the Aerodyne high-resolution time-of-flight aerosol mass spectrometer in line with a thermodenuder (TD-HR-TOF-AMS), which measures the mass fraction remaining (MFR) of OA at different temperatures [146,192-194]. An evaporation kinetic model was applied for the data in the Beijing and Gucheng while an empirical method was used for the data in Guangzhou and Dongying to drive the volatility distributions of primary OA factors obtained by the PMF method. The distributions shown in Fig. S21 have 5 bins with C^* from 10^{-2} to 10^2 μg m^{-3}. Contributions from low-volatility bins were summed to the lowest bin with C^* of 10^2 μg m^{-3}.

Other measurements used in model performance evaluations. The surface concentrations of OH and HONO from individual campaigns from literature are also collected for the model evaluation in Table S6. This data set contains 9 surface measurements of OH and 23 measurements of HONO from 2014 to 2019. In these studies, OH was measured by laser-induced fluorescence (LIF) or fluorescence assay by gas expansion (FAGE) instruments [195-204]. HONO was measured by instruments included long path absorption photometer (LOPAP), Ambient Ion Monitor (AIM), and other analyzers [205-215].

Note S3. Model-observation Comparisons and Model Consistency

Population weighting. We applied the population data from the Gridded Population of the World (https://sedac.ciesin.columbia.edu/data/collection/gpw-v4) dataset for 2015 to derive the population-weighted POA and SOA concentrations. The trends of population-weighted and arithmetic-mean concentrations of POA and SOA in China are similar (Fig. S22). The population-weighted POA and SOA annual mean concentrations decrease by 54% and 25% from 2013 to 2020, while the arithmetic means decrease by 47% and 24%, respectively. The population-weighted concentrations are higher because high anthropogenic emissions usually occur in areas with high population density.

Comparisons of OC. The model reproduces the observed declining trends of annual OC concentrations in NCP (*n*=34) from 2017 to 2020 and YRD (*n*=6) from 2015 to 2020 (Fig. S5*A-B*). In NCP, the OC concentration shows relatively flattened concentrations between 2018 and 2019 compared to faster decreases in concentrations from 2017 to 2018 and from 2019 to 2020. Seasonal mean OC concentrations in NCP are high in winter and low in summer as is consistent with the greater residential emissions of S/LVOC in winter (Fig. S1). Indeed, the observed annual OC concentrations show a larger decrease by about 40% in winter than in other seasons (33%, 30%, and 13% for autumn, spring, and summer, respectively) from 2017 to 2021, explained by the greater wintertime residential emission reduction. Similar seasonal trends have been also observed at the research site in Beijing during 2013-2020.The model shows a good performance of annual mean OC concentrations in the NCP region with a normalized mean bias (NMB) of 0.02 and a Pearson's correlation coefficient (*r*) of 0.67 (Fig. S5*C*). Comparing the observed and simulated decreases of annual mean OC concentrations from 2019 to 2020 in NCP further shows both observation and simulation show a larger decrease in southern Hebei province compared to other areas (Fig. S17). This result suggests that the model can capture the spatial distribution of the decreases in OC concentrations in NCP. In YRD, the OC concentration decreased consistently from 2015 to 2020. Similar to NCP, the decrease is generally faster from 2016 to 2018 and slower from 2018 to 2019. The model captures the OC concentrations with an NMB of -0.11 (Fig. S5*D*). The *r* of 0.15 however is much lower than NCP due to the overestimation at the Jiangsu sites and underestimation at the Zhejiang sites. The compensating error in YRD OC concentration suggests underrepresented local emissions in specific areas although the regional amounts are captured.

Comparisons of POA and SOA. The evaluations against the mean concentrations of POA and SOA from individual campaigns are shown in Fig. 1*E-F* and S5*E-F* and Tables S2 and S3. For evaluating the whole measurements, the model shows a good performance for both POA and SOA with NMBs of -0.05 and -0.26, respectively. The *r* for POA and SOA are 0.87 and 0.54, respectively. Although our model is unable to reproduce some of the high concentrations, those measurements were taken either at sites where biomass burning or residential coal burning plumes contributed predominantly to POA or when so-called "aqueous processes" occurred to cause enhanced SOA formation during severe winter-haze events. The former is difficult to be captured by the nationwide inventory [38], while the mechanisms of the latter remain unclear enough for CTMs to parameterize [216,217]. Comparing the model performance between regions shows relatively better performance in NCP, YRD, and PRD, which are the most populated regions in China, with NMBs in the range of -0.34 to 0.13 for both POA and SOA. The NMBs of POA and SOA in NW, however, are over -0.50, which may result from the uncertainties in emission inventory. Evaluating the interannual variations of potential model biases for POA and

SOA shows the NMBs in the range of -0.27 to 0.17 and -0.47 to -0.02, respectively. The variations of NMB for POA and SOA suggest that the declining trends of POA and SOA and the increasing SOA contributions shall not be distorted by the model biases (Fig. 1*C*–*D*).

The comparisons between the sum of HOA, CCOA, COA, and BBOA to the modeled POA and the sum of OOAs to the modeled SOA are meaningful for the following reasons. First, the four primary PMF factors show mass spectra and mass ratios of OM:OC similar to freshly emitted OA from transportation, residential or industrial combustion, cooking, and biomass burning at ambient loading levels [134,218], which covers the main sources of primary emissions in China. The model in this study considered full-volatility-range organic emissions from all these primary sources except cooking and applied volatility-based partitioning to determine the portion of organics (S/LVOC) that can remain in the particle phase after being emitted (so-called the modeled POA; labeled as the EPOA tracer). An OM:OC ratio of 1.4 is used for EPOA which is consistent with the ratios of PMF primary factors (1.2-1.6). In this regard, we think it is reasonable to use the comparison between the sum of PMF primary factors to the modeled POA as a validation of the model settings of the S/LVOC emissions and their volatility distributions as long as cooking is not a major contributor. Cooking emissions are lack of good constraints on a national level. The PMF results indicate 8-33% of the OA mass as COA with insignificant yearly changes in cities [121]. We expect <4% of the populated-weighted OA mass from cooking by assuming a COA mass fraction of 15% in model grids with population densities of >1000/km^2 and little interannual variations. Therefore, the overlooked COA should not affect much the modeled POA mass and trends herein. We kept COA in the observation dataset because the PMF analysis has been proven to robustly separate primary factors from oxidized “secondary” factors while individual primary factors may not be well resolved depending on instrument tuning and analysis skills. Nevertheless, for the model-observation comparison of POA in Fig. S5*E*, if COA is excluded from the observation data, the comparison gives a r of 0.85 and a NMB of 0.25, still supporting a good model representation of S/LVOC. Other evidence to support the good model representation of S/LVOC include the consistent China’s emissions with other inventories (Fig. S3), the consistent estimation of US emissions with the findings of Pye et al. (https://doi.org/10.23719/1527956) (Fig. S4), and the roughly reproduced total mass fraction of volatility bins below C^* of 1 μg m^{-3} (predominantly in the particle phase) although the fraction of individual bin has not been well captured (Fig. S21). Second, the PMF OOA factors are identified by mass spectra similar to SOA and much greater OM:OC ratios (1.8-2.2) than the ratios of primary factors (e.g., [134]). These OOA factors may represent SOA from various precursors with different photochemical age as well as aqueous SOA [93,121]. In the model, SOA is the sum of biogenic SOA, aromatic SOA, IVOC SOA, S/LVOC SOA (labeled as the OPOA tracer), and key aqueous SOA, covering almost all important precursors as well as the oxidation and aqueous processes. An OM:OC ratio of 2.1 is applied to the modeled SOA, which is again consistent with the PMF findings. We think the comparisons between the sum of PMF OOAs to the modeled SOA along with the model-observation comparison of precursor concentrations validate general model skills on the SOA precursor emissions and chemical processes.

Comparisons of SOA precursors. Ambient measurements of total I/SVOC and their volatility distributions are quite limited in China [138,146,186,188]. The simulated surface concentrations of IVOC agree with the observations in Shanghai and Guangzhou except for some underestimation in winter (Fig. S19*B*). In terms of the volatility distributions of IVOC, the observations show greater contributions of the high-volatility bin with C^* of 10^6 μg m^{-3} that has little impact on SOA except for the urban Shanghai site that has a higher fraction in the low-volatility bin (Fig. S20). The different

volatility distributions of IVOC between sites may result from the differences in local sources. Uncertainties in emission and differences in volatility distributions of IVOC between sources lead the observed IVOC volatility distributions to be hard to capture well [18,26]. For SVOC, small NMB values and good correlations between the modeled and observed campaign-mean POA concentrations from 126 field measurements in China indicate a reasonable estimation of SVOC. The observed volatility distributions of POA also show differences between sites that may result from differences in local sources (Fig. S21). For example, the fraction of the low-volatility bin with C^* equal to or less than 10^{-2} μg m^{-3} is much higher in Dongying (a receptor site in NCP) and Guangzhou (an urban site in southern China) compared to the urban and rural sites in NCP. The model generally reproduces the observed volatility distributions of POA but overestimates the fractions of the bins with C^* of 10^1 and 10^{-1} μg m^{-3}.

Other comparisons. The model performances for OH and HONO are significantly improved by the updated parameterizations in our model compared to the default version. As shown in Table S6, the NMB and root mean square error (RMSE) of HONO in our model decrease from -0.49 and 0.84 to -0.14 and 0.63, respectively. This improvement affects OH simulation mostly in winter with the NMB and RMSE of the diurnal maximum of OH concentration decreasing from -0.73 and 2.28 to -0.25 and 1.34, respectively. The change in the model performance for nitrate is minor in the updated model. Additionally, the underestimation of sulfate is largely reduced in our model compared to the default version with the improved OH simulation and added heterogeneous formation pathway.

Model Consistency with Literature*.* Table S9 summarize the global burdens of OA reported in different GEOS-Chem studies. The modeled POA under non-volatile POA treatment are much greater than the modeled POA under semi-volatile treatment. This is because the quartz filter based POA emissions represent organic emissions over a range of volatilities. Some semi-volatile portion of this emission remain in the particle phase during the atmospheric dilution but some are gas precursors that may further produce SOA (S/LVOC-SOA). Advanced emission profile measurements suggest a scaling factor of 1.0 (1.4 for gasoline) to the filter-based POA emissions to represent the total (gas+particle) emissions of S/LVOC of anthropogenic sources [28,219]. Therefore, the non-volatile POA treatment overestimates the particulate organic emissions. This is confirmed by significant greater modeled POA than the AMS-derived POA in previous studies [220,221].

The question is what fraction should be positioned as freshly emitted POA. Brew et al. [2023] found EPOA under non-volatile POA treatment reproduce better the AMS-derived POA, and the rest (the hydrophilic portion of non-volatile POA; also labeled as OPOA), if considered as additional S/LVOC-SOA, leads to a match between modeled SOA and AMS-derived OOA. This indicates that a majority of the filter-based POA emissions should be gaseous S/LVOC in the "KORUS-AQ" region [222] and that the Hodzic representation on S/LVOC- and IVOC-SOA is insufficient. In this study, we developed full-volatility-range organic emissions. The S/LVOC-SOA and IVOC-SOA are parameterized on the volatility distributions of their precursor emissions (8 bins total). With the revised model scheme, a high fraction of the filter-based POA emissions remain as EPOA in highly polluted anthropogenic source regions (Fig. *S23A*), which explains why the model can reproduce high concentrations of AMS-derived POA in China. The EPOA/(EPOA+OPOA) fractions decrease in downwind and remote regions (Fig. S23*B*).

Table S9 suggest that S/LVOC-SOA+IVOC-SOA in the revised scheme is comparable to OPOA+SVOC-SOA+IVOC-SOA in the Hodzic scheme with non-volatile POA treatment, indicating

a consistent SOA budget between our study and Brew et al. [2023]. Previous studies might have misplaced some categories of OA and the standard complex scheme is certainly outdated.

On the total amount of OA, our study is consistent with previous model studies that were evaluated extensively by the observations [59,223]. We provide a bottom-up volatility-based simulation scheme to capture the underestimated anthropogenic contributions with careful evaluations for China. Our treatment of S/LVOC-SOA (although labeled as OPOA in this study) and IVOC-SOA as the modeled SOA is constrained by the AMS-derived OOA and ambient flow-tube observations on SOA potential [224]. Globally, well-constrained emission inventories over the entire volatility range along with improved representation of their SOA formation are needed to reproduce the spatial variabilities of OA.

Supplementary Figures

Figure S1. The annual-mean OC concentrations in different seasons (DJF: winter; MAM: spring; JJA: summer; SON: fall) (*A*) in NCP obtained from the long-term continuous offline measurements at 34 sites of the national network and (*B*) in Beijing obtained from long-term online measurements at a research roof site in the campus of Peking University.

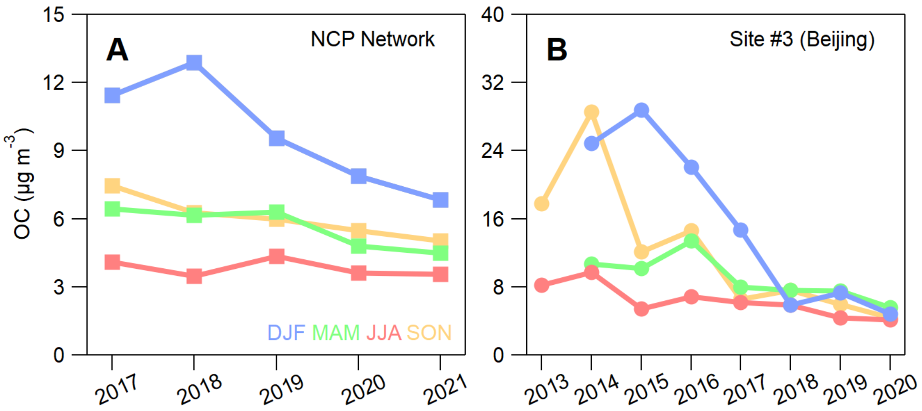

Figure S2. (*A*) Anthropogenic emissions of OA precursors from VOC, IVOC, and S/LVOC in China from 2013 to 2020. (*B–D*) The annual mean emission compositions of NMVOC, IVOC, and S/LVOC grouped by species or C^* at 298 K in China. Aromatic compounds, GLY, MGLY, isoprene, and terpenes are typical OA precursors, which are about 34-37% of NMVOC and are outlined by light green wedges in panel B. In panel C, the light green wedges highlight the volatility bins that have the lowest vapor pressure and highest SOA formation potential. In panel D, the light green wedges highlight the volatility bins that are primarily distributed in the particle phase.

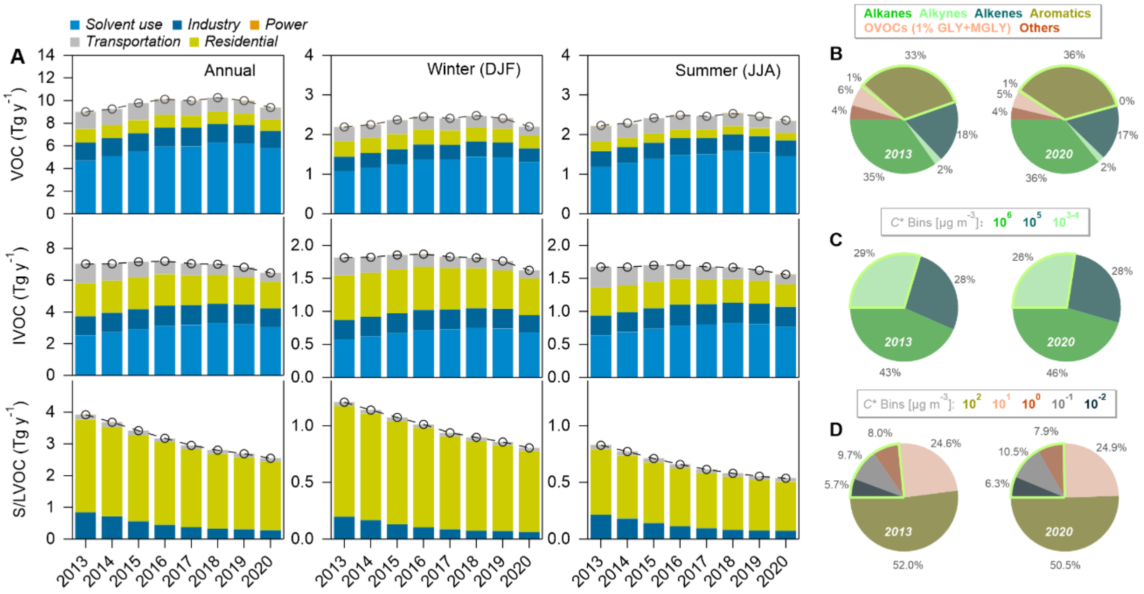

Figure S3. Comparisons of China's anthropogenic (*A*) IVOC and (*B*) S/LVOC emissions among inventories. The IVOC and S/LVOC emissions estimated by Zheng et al. [225] is for 2013-2019, and those estimated by Wu et al. [226] is for 2016. Note that the long-term emissions from Zheng et al. [225] based on the method developed by Chang et al. [26]. The transportation IVOC emissions from Zhao et al. [227] and Wang et al. [228] for 2017 and 2019, respectively, are also used in the comparisons of transportation sector. For Wang et al. [228] only considering on-road emissions, we estimate corresponding transportation emissions by assuming on-road emissions contribute 53% of IVOC in the transportation sector [227].

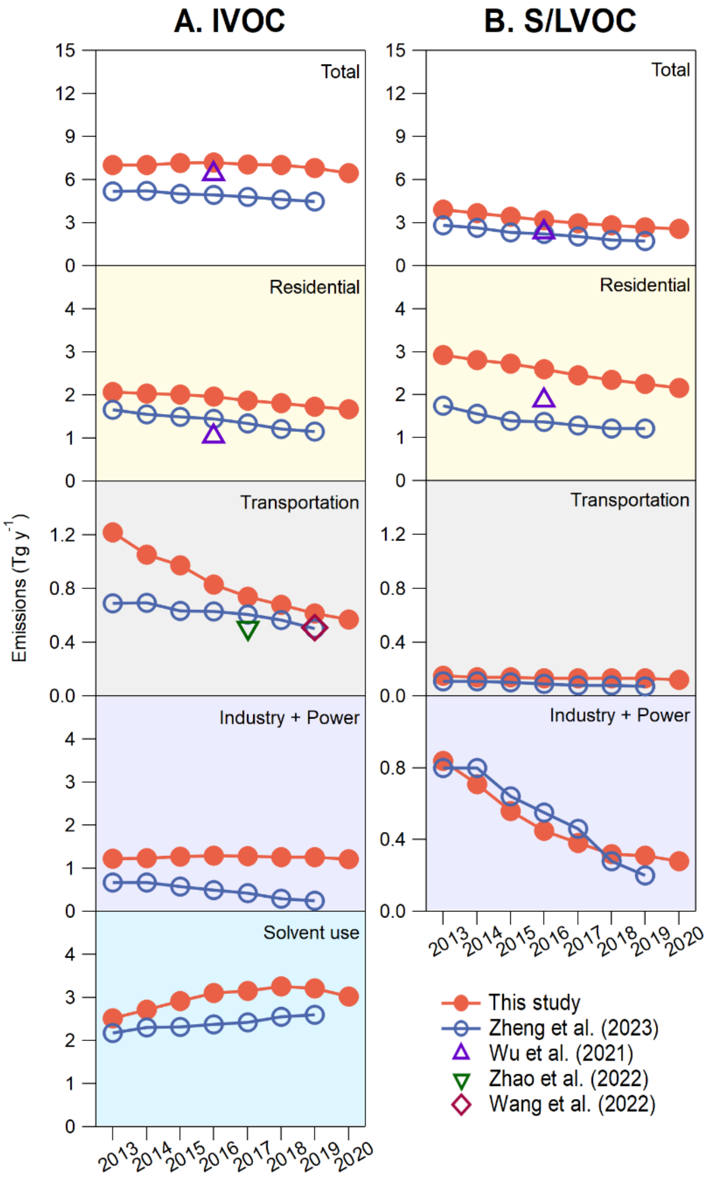

Figure S4. Open biomass burning emissions of (*A*) IVOC and S/LVOC in China from 2013 to 2020. (*B*) China's emissions of OA precursors in 2017 compared to the emissions in the United States. The IVOC and S/LVOC emissions in the United States are estimated using the same method as China with NMVOC and OC emissions from CEDS [11]. The emissions from CEDS working sectors are reclassified to fuel-based sectors like MEIC and then scaled by corresponding emission scalars for IVOC and S/LVOC as described in Section S1. The other IVOC and S/LVOC emissions in the United States in 2017 calculate from a species-level inventory developed by Pye, et al. [229] are also shown for comparison (Data is archived at https://doi.org/10.23719/1527956).

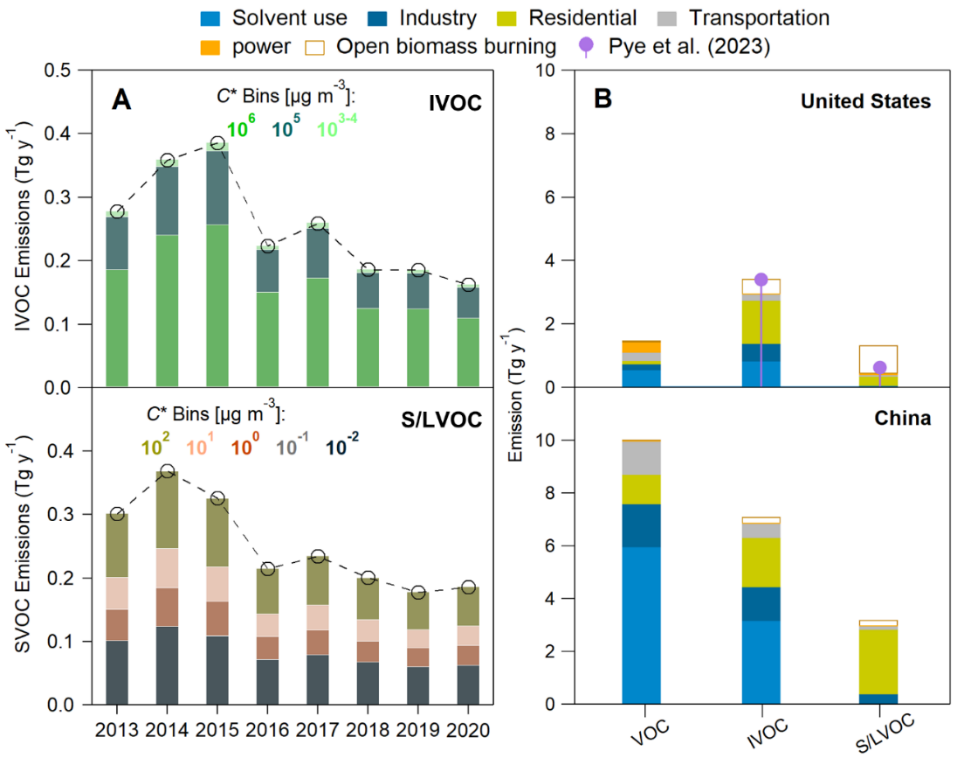

Figure S5. Observations compared to model simulations in China. (*A-B*) Annual-mean OC concentrations derived from continuous measurements at 34 sites in a recently-developed national monitoring network for $PM_{2.5}$ chemical composition in NCP and at 6 provincial monitoring sites in YRD. Only the sites with annual data coverage of >75% were used to calculate the annual-mean concentrations. In YRD, 1 or 2 sites did not meet the criteria for some years. The actual sites in each year are listed in gray in panel *B*. Error bars in panels *A* and *B* represent the standard deviations of the mean concentrations of sites in NCP and the ranges of the mean concentrations of sites in YRD, respectively. (*C-D*) Yearly averages of OC concentrations obtained from the NCP (*n*=34) and YRD (*n*=6) network sites (including low data-coverage ones as the model simulations matched with the measurement periods) as well as the literature data from different research sites in the two regions. (*E-F*) Mean concentrations of POA and SOA from individual campaigns from 2013 to 2020 in China colored by different seasons (DJF: winter; MAM: spring; JJA: summer; SON: fall).

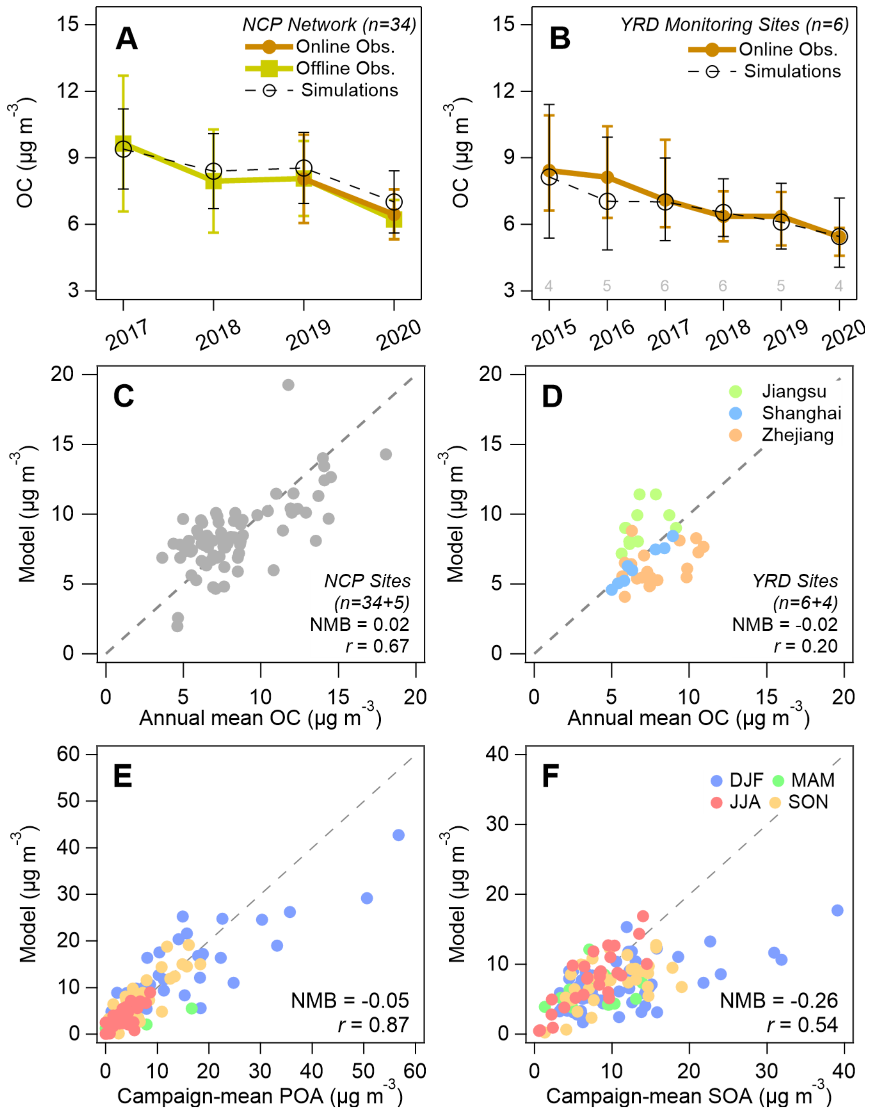

Figure S6. The 2020−2013 annual emission changes of POA and SOA precursors from (*A*) anthropogenic and (*B*) biogenic sources. Units are Gg yr^{-1}. The coastline boundaries in the map are originated from Natural Earth free vector map data (https://www.naturalearthdata.com/). The administration boundaries are originated from National Earth System Science Data Center (https://www.geodata.cn).

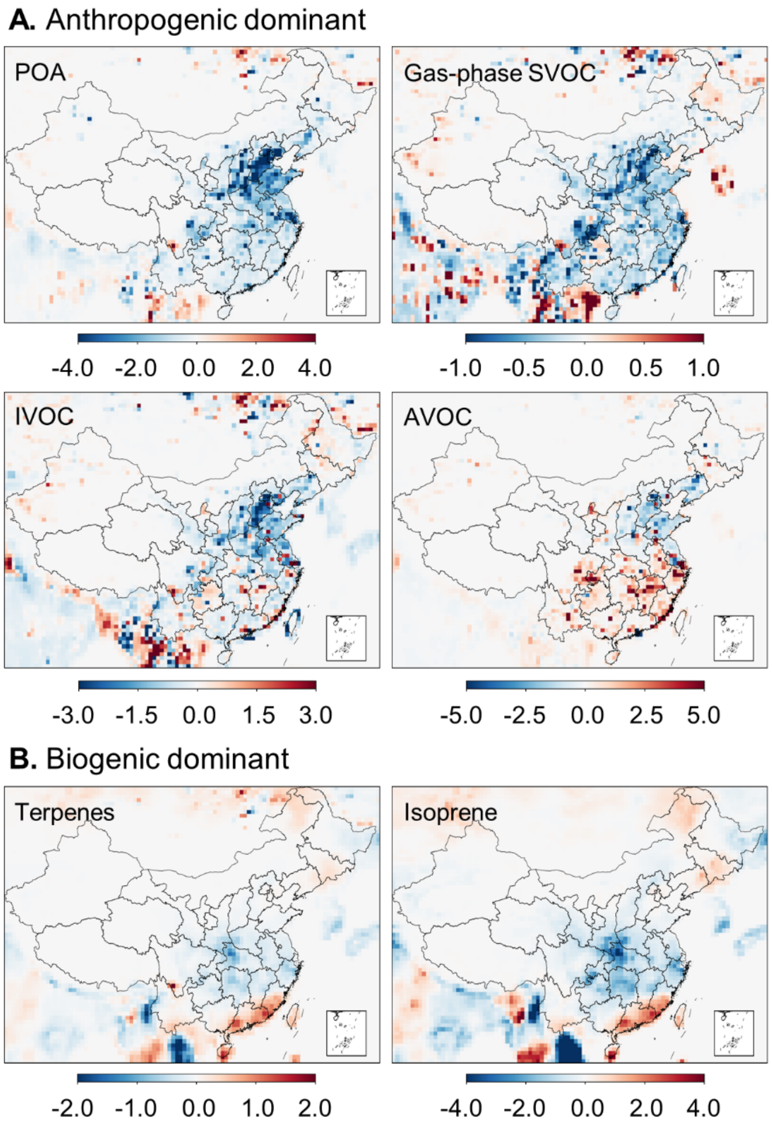

Figure S7. Forest fire influence in Sichuan Province in 2020. (*A*) The 2020−2013 changes of annual emissions of BC from open biomass burning. (*B*) The 2020−2013 changes of annual emissions of POA from open biomass burning. The coastline boundaries in the map are originated from Natural Earth free vector map data (https://www.naturalearthdata.com/). The administration boundaries are originated from National Earth System Science Data Center (https://www.geodata.cn).

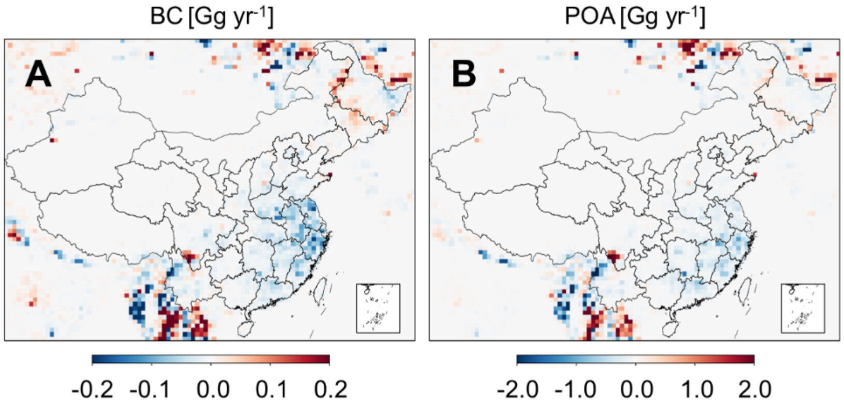

Figure S8. (*A-B*) The mass fraction of POA in OA in 2020 and in 2013 in China. (*C-D*) The 2020-2013 difference of the mass fraction of POA and SOA in OA in China. The coastline boundaries in the map are originated from Natural Earth free vector map data (https://www.naturalearthdata.com/). The administration boundaries are originated from National Earth System Science Data Center (https://www.geodata.cn).

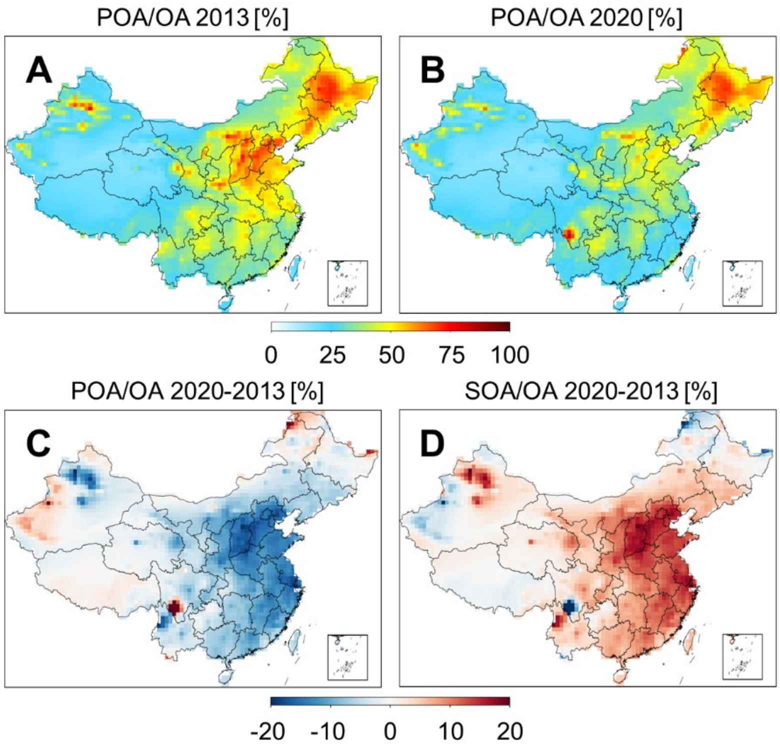

Figure S9. Annual changes of precursor emissions compared to the changes of corresponding types of OA concentrations.

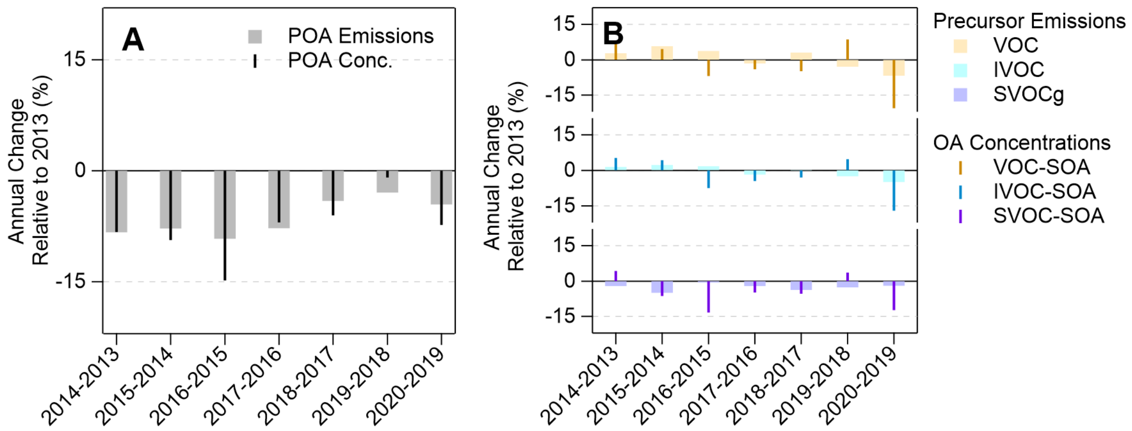

Figure S10. Contributions of biomass burning and biogenic emissions to the emission-driven changes of (*A*) POA and (*B*) SOA relative to 2013.

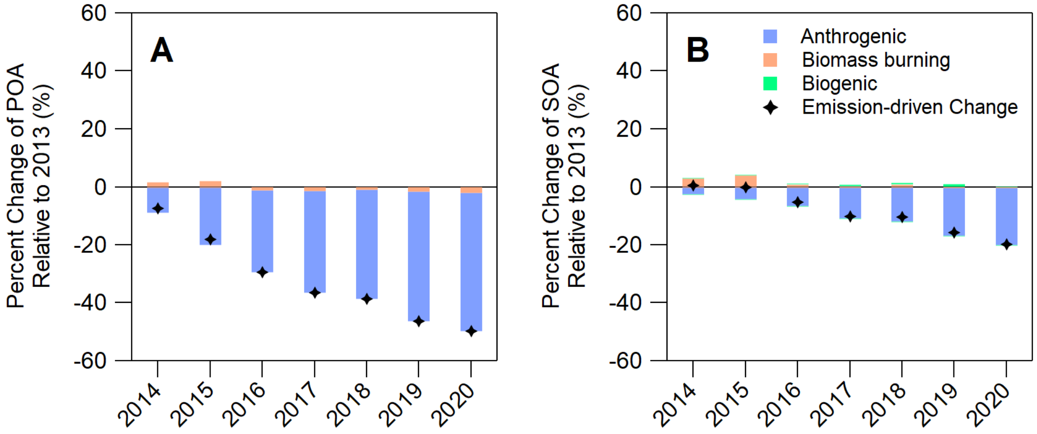

Figure S11. Normalized changes of (*A*-*B*) annual mean concentrations of emission-fixed POA and SOA and (*C*-*D*) annual mean meteorological factors. The emission-fixed annual mean POA and SOA concentrations are obtained from simulations with fixed anthropogenic emissions of 2013 to isolate the meteorology-driven interannual variations (i.e., 2013 from Base and 2014-2020 from CTL_ALLEMIS simulation (Table S8)). Meteorological inputs are from MERRA2. The stagnation days are calculated from 10-m wind speed, PBLH, and precipitation [230]. The *r* values for the correlations between the normalized changes of POA and SOA and the normalized changes of meteorology are shown.

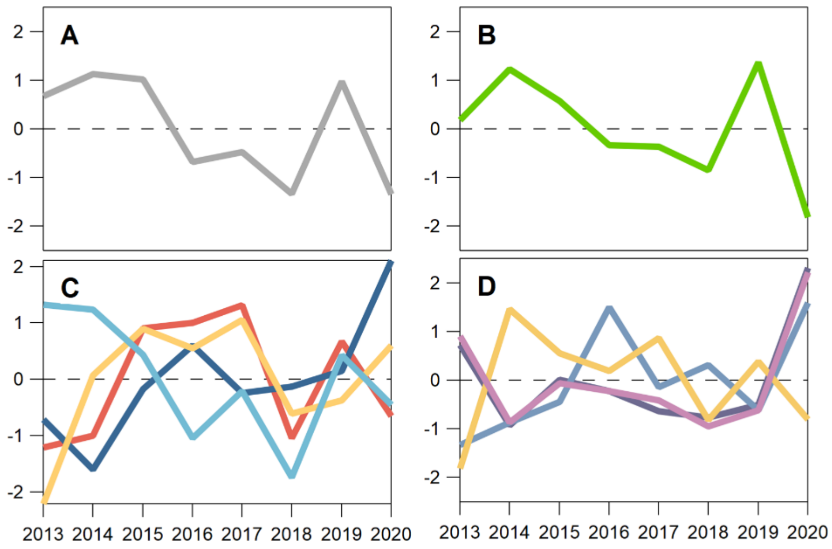

r between normalized trend	POA (emission fixed)	SOA (emission fixed)
Surface temperature	0.07	0.16
Pressure	-0.22	-0.18
Relative humidity	-0.66	-0.73
Stagnation days	0.86	0.66

r between normalized trend	POA (emission fixed)	SOA (emission fixed)
PBLH	0.37	0.51
Precipitation	-0.81	-0.75
Cloud fraction	-0.33	-0.61
Cloud optical thickness	-0.30	-0.60

Figure S12. Scatter plots of meteorology-driven annual concentration changes derived from CTM and predicted annual changes by the random forest model (A) with and (B) without using concentrations as an input parameter in the hold-out validation data set.

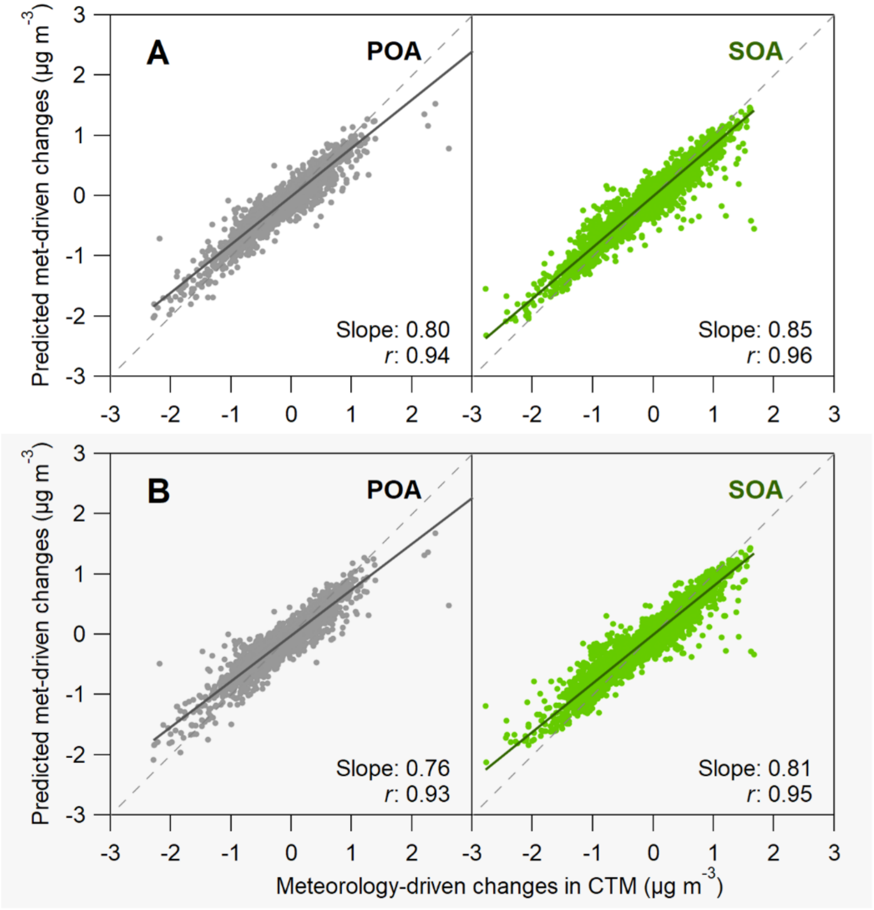

Figure S13. (A) Feature importance and (B) absolute SHAP values of meteorological inputs to the meteorology-driven interannual variations of POA and SOA resolved by random forest model analysis with (left) and without (right) using concentrations as an input parameter. The concentrations of POA or SOA (i.e., CONC) used in RF are the model results under the fixed-2013-emission scenario.

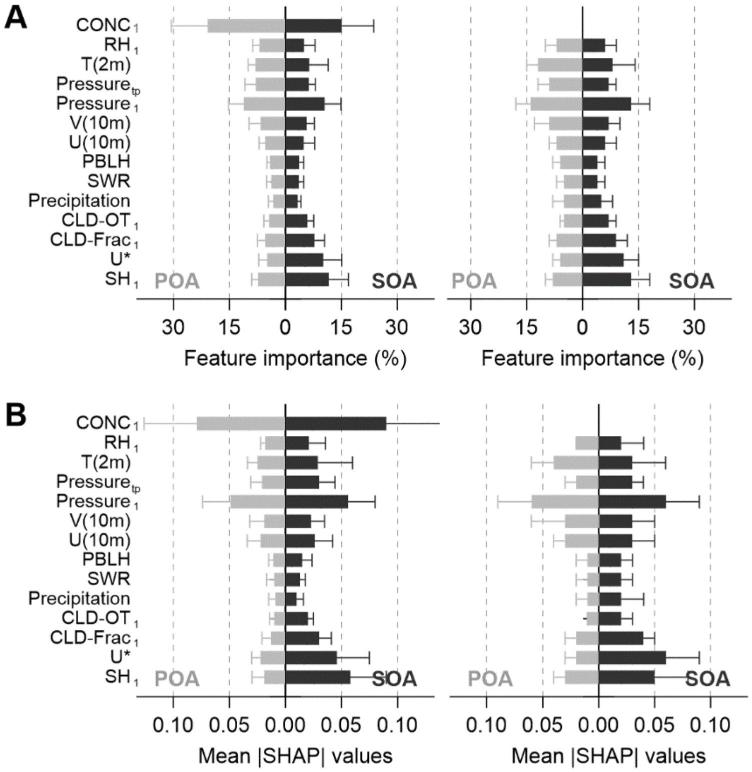

Figure S14. The 2020-2013 difference of surface concentrations of NO_x, SO_2, non-OA-precursor VOC, hydroxyl radical, ozone, and aerosol liquid water content (ALWC) in China. The coastline boundaries in the map are originated from Natural Earth free vector map data (https://www.naturalearthdata.com/). The administration boundaries are originated from National Earth System Science Data Center(https://www.geodata.cn).

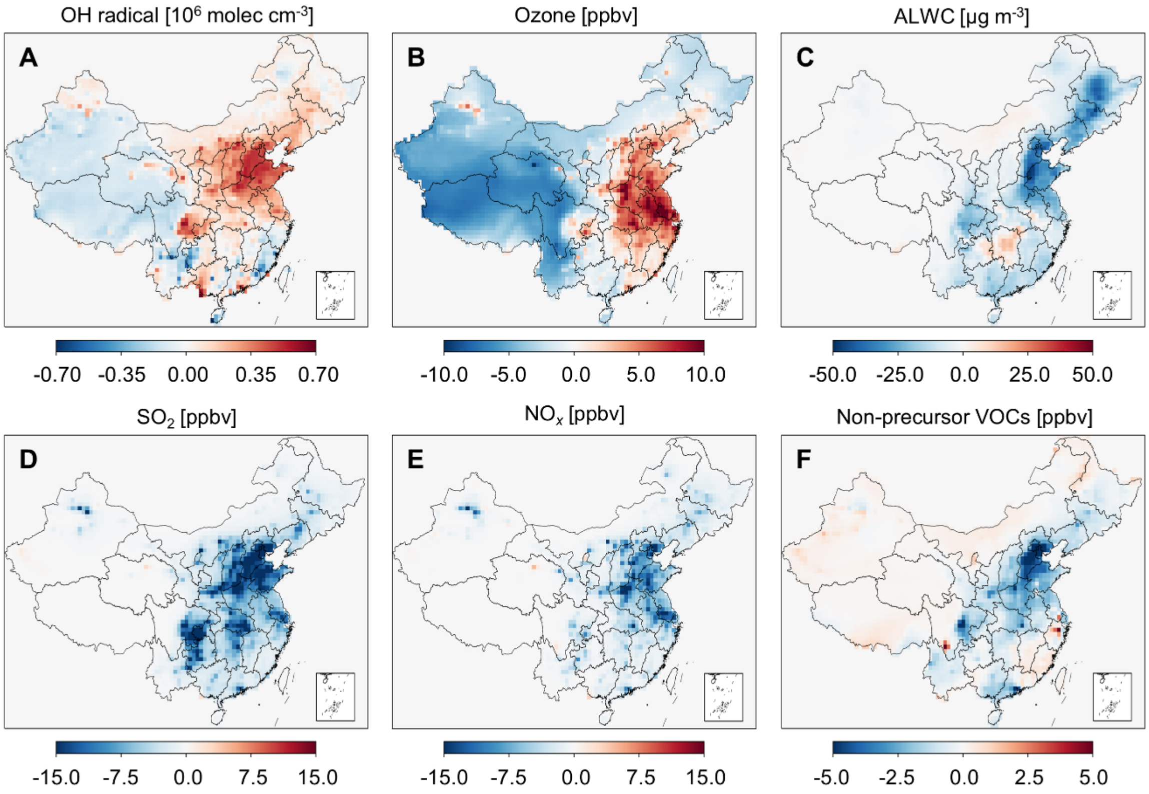

Figure S15. The changes of SOA concentrations led by chemical drivers during the two action periods. The percent values in black represent the changes of population-weighted SOA concentration relative to 2013. The colored percent values in the lower left of each panel represent the contributions of emission controls on SO_2, NO_x, and other non-OA-precursor pollutants to the SOA changes. We summed up the changes of SOA tracers related to gas-phase oxidation processes to represent the impacts of oxidant levels and the changes of SOA tracers related to heterogeneous uptake processes to represent the impacts of ALWC as well as aerosol *p*H. The coastline boundaries in the map are originated from Natural Earth free vector map data (https://www.naturalearthdata.com/). The administration boundaries are originated from National Earth System Science Data Center (https://www.geodata.cn).

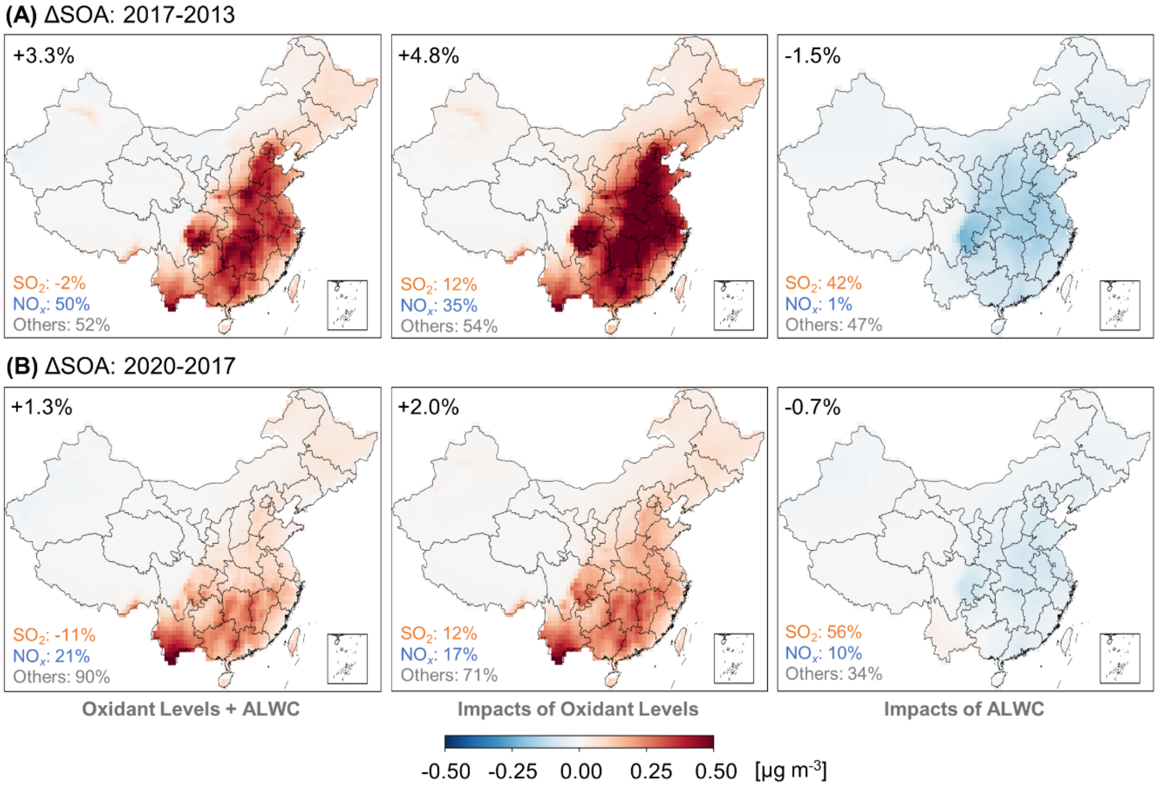

Figure S16. Emission- and meteorology-driven monthly variations of surface (A) POA and (B) SOA concentrations in NCP in 2017 relative to 2013.

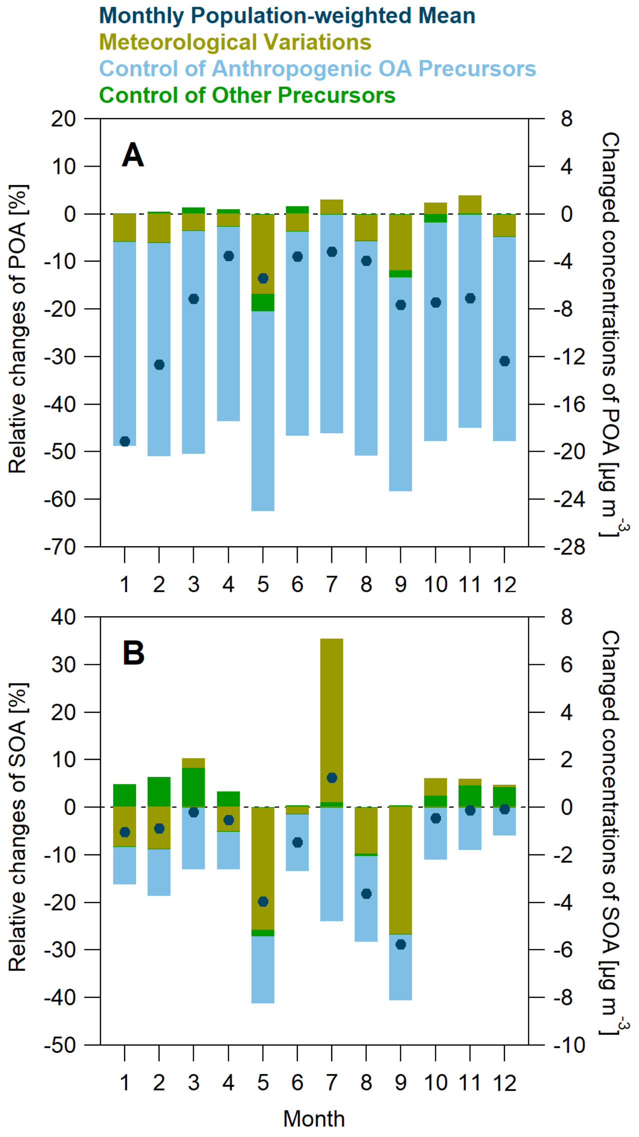

Figure S17. Observed and simulated 2020-2019 decreases of annual mean OC concentrations in NCP in China. Circles represent the observed OC decrease at the monitoring sites. The administration boundaries are originated from National Earth System Science Data Center(https://www.geodata.cn).

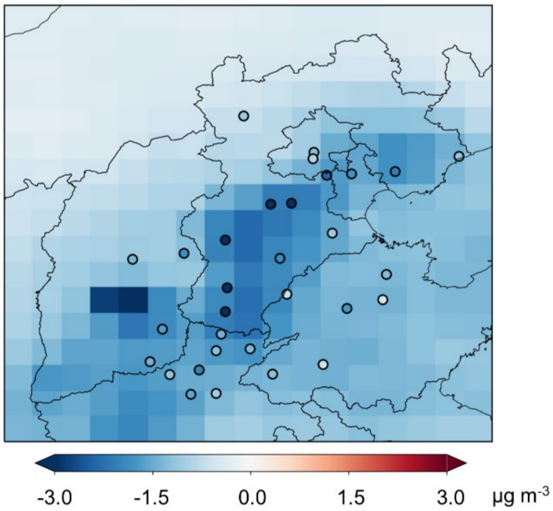

Figure S18. Emissions of OA precursors in India and Southeast Asia area in 2017. These emissions are estimated using the same method as China but on the basis of NMVOC and OC emissions from the global CEDS inventory instead of MEIC [11]. The emissions from CEDS working sectors are regrouped the sectors used in MEIC to compare with China's emissions. The Southeast Asia area includes Brunei, Cambodia, East Timor, Indonesia, Laos, Malaysia, Myanmar, Philippines, Singapore, Thailand, and Vietnam.

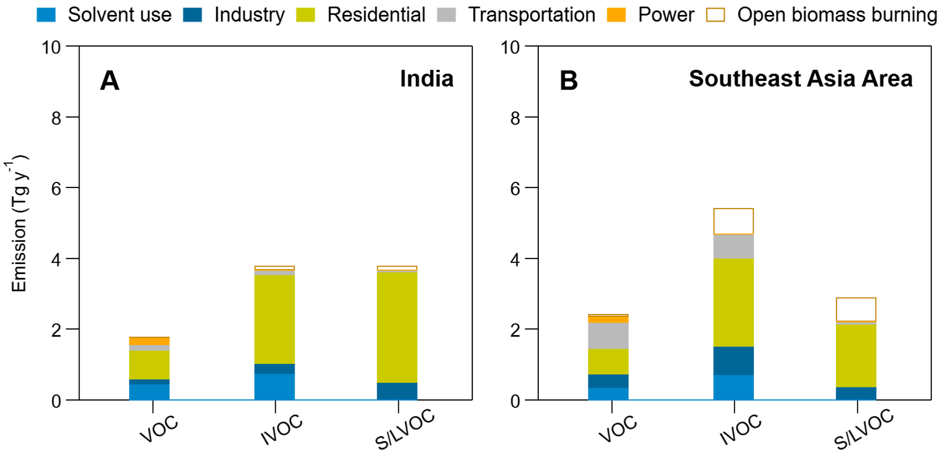

Figure S19. Scatter plots of the observed and simulated campaign-average mixing ratios of IVOC. Error bars represent the standard deviations of the measurements in each campaign. The coastline boundaries in the map are originated from Natural Earth free vector map data (https://www.naturalearthdata.com/). The administration boundaries are originated from National Earth System Science Data Center (https://www.geodata.cn).

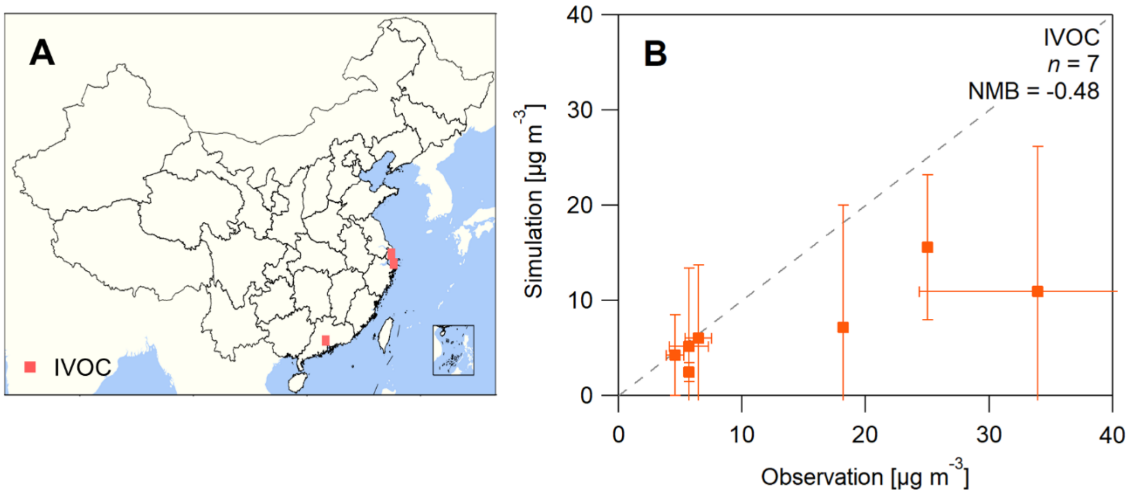

Figure S20. Comparisons of the observed and simulated campaign-mean volatility distributions of ambient IVOC in Shanghai (SH) and Guangzhou (GZ).

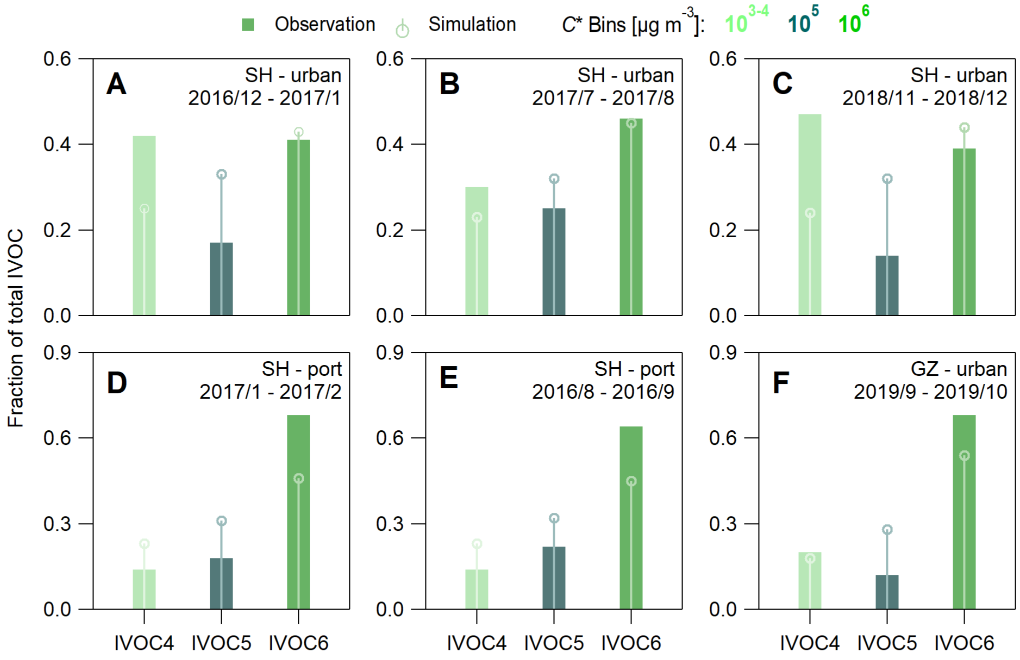

Figure S21. Comparisons of the observed and simulated campaign-mean volatility distributions of ambient POA in China.

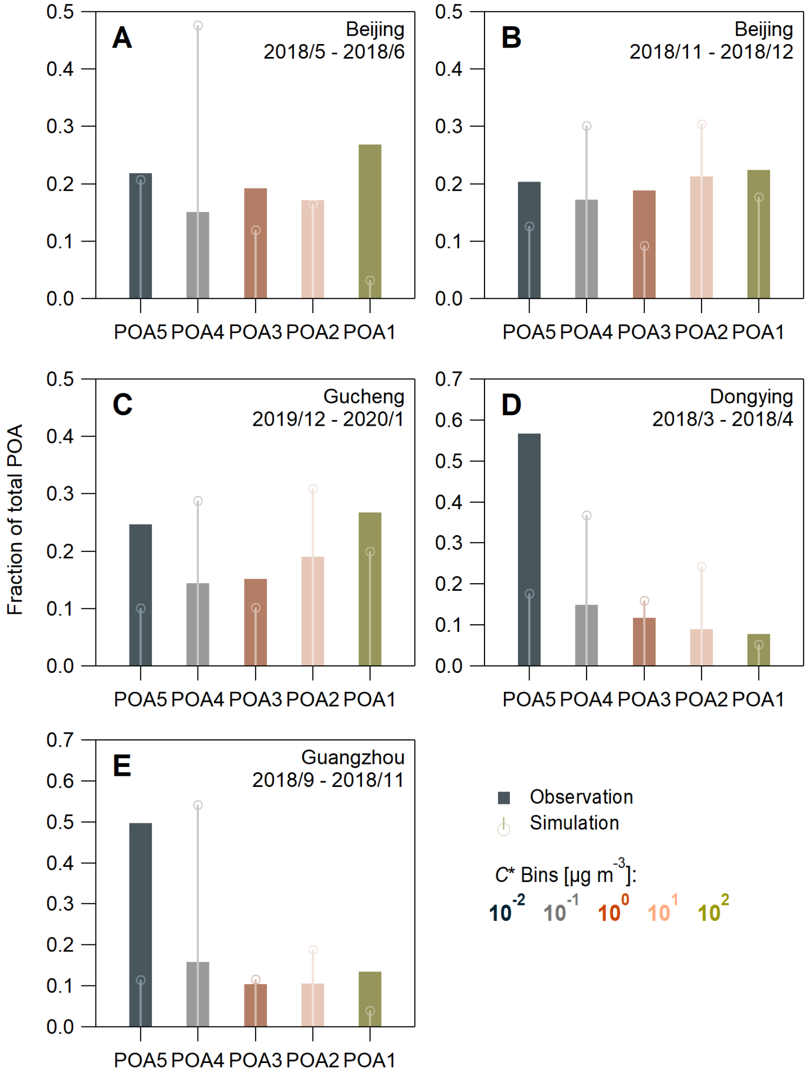

Figure S22. The population-weighted and arithmetic-mean annual concentrations of POA and SOA in China.

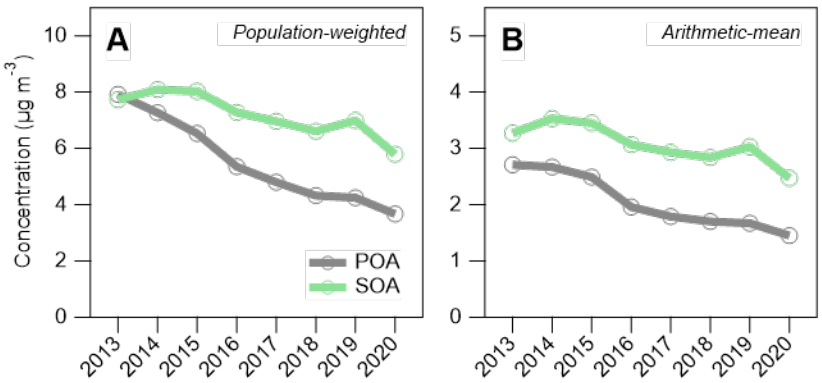

Figure S23. The modeled EPOA / (EPOA + OPOA) at (A) surface and (B) at the altitude of 3 km in 2013. EPOA and OPOA are the tracers that represent the modeled POA and S/LVOC-SOA, respectively. The coastline boundaries in the map are originated from Natural Earth free vector map data (https://www.naturalearthdata.com/). The administration boundaries are originated from National Earth System Science Data Center (https://www.geodata.cn).

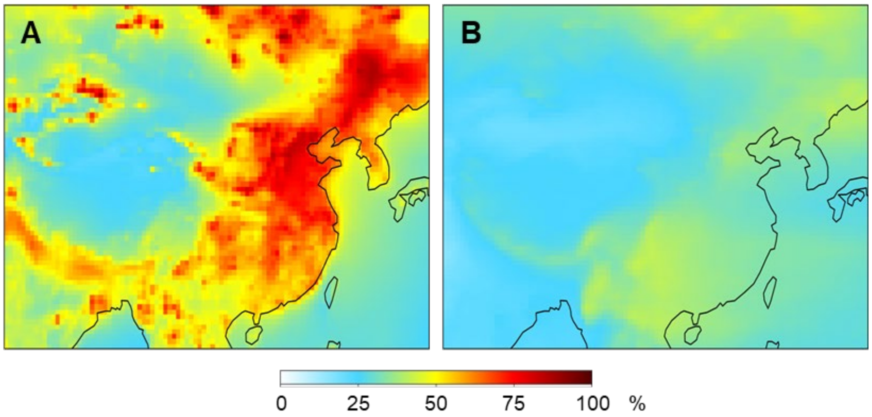

Supplementary Tables

Table S1. List of the observed and simulated campaign-mean concentrations of OA, POA, and SOA. The observed POA and SOA concentrations were derived from positive matrix factorization (PMF) analysis on online measurements by aerosol mass spectrometers. Temporally overlapped measurements from literature are not included in Fig. S4 but used for observation data validation.

No.	City	Region	Longitude	Latitude	Sampling Period	Observations [μg m^{-3}]			Model Simulations [μg m^{-3}]			Ref.
						OA	POA	SOA	OA	POA	SOA	
1	Beijing	NCP	116.31	39.99	1/23/2013-3/2/2013	29.70	14.90	14.80	32.00	25.28	6.72	76
2	Nanjing	YRD	118.73	32.21	1/4/2013-1/31/2013	26.26	14.11	11.89	35.66	20.37	15.30	77
3	Hongkong	PRD	114.17	22.32	3/7/2013-5/15/2013	12.80	7.90	4.90	7.86	2.06	5.80	78
4	Hongkong	PRD	114.17	22.32	5/16/2013-7/19/2013	7.90	5.60	2.20	3.62	0.82	2.80	78
5	Nanjing	YRD	118.76	32.08	6/1/2013-6/15/2013	15.40	3.30	11.20	13.88	5.47	8.41	79
6	Xianghe	NCP	116.96	39.80	6/9/2013-7/9/2013	18.10	8.60	9.50	21.62	8.92	12.70	80
7	Nanjing	YRD	118.77	32.05	8/1/2013-8/31/2013	10.30	2.73	6.86	12.39	4.32	8.08	81
8	Menyuan	OTR	101.26	37.61	9/5/2013-10/15/2013	4.90	0.80	4.10	0.93	0.28	0.65	82
9	Beijing	NCP	116.37	39.97	10/1/2013-11/15/2013	29.51	14.83	14.67	23.69	14.99	8.70	83
10	Qingdao	NCP	120.47	36.10	11/1/2013-11/30/2013	10.43	5.57	4.86	9.39	4.48	4.91	84
11	Hongkong	PRD	114.17	22.32	9/3/2013-12/31/2013	15.10	6.30	8.80	11.93	3.53	8.40	85
12	Nanjing	YRD	118.76	32.08	10/15/2013-10/30/2013	22.30	7.20	13.10	16.69	8.91	7.78	79
13	Lin'an	YRD	119.73	30.30	11/16/2013-12/18/2013	29.00	13.40	15.70	24.77	12.40	12.37	86
14	Nanjing	YRD	118.77	32.05	12/1/2013-12/31/2013	38.40	15.74	22.66	34.88	21.63	13.25	87
15	Dongguan	PRD	113.75	23.03	12/12/2013-1/1/2014	23.30	7.36	15.75	22.27	10.01	12.26	84
16	Beijing	NCP	116.37	39.97	12/19/2013-1/2/2014	36.30	22.30	13.80	19.57	16.40	3.17	88
17	Beijing	NCP	116.38	40.00	1/9/2014-1/26/2014	43.00	35.67	7.40	32.26	26.20	6.06	89
18	Lanzhou	NW	103.85	36.05	1/10/2014-2/4/2014	29.33	18.41	10.91	8.01	5.57	2.44	90
19	Shijiazhuang	NCP	114.54	38.03	1/11/2014-2/18/2014	89.00	64.97	24.03	43.10	34.53	8.57	91
20	Baoji	NW	107.14	34.35	2/26/2014-3/27/2014	29.70	16.63	13.07	10.55	5.52	5.03	92
21	Beijing	NCP	116.37	39.97	6/3/2014-7/11/2014	18.10	7.78	10.32	19.32	6.66	12.66	93
22	Xinzhou	OTR	112.12	38.07	7/17/2014-9/5/2014	11.72	2.20	9.50	9.25	3.26	5.99	94
23	Beijing	NCP	116.37	39.97	10/1/2014-11/15/2014	25.55	10.82	14.73	24.11	14.40	9.72	83
24	Beijing	NCP	116.03	39.60	10/22/2014-11/11/2014	29.68	11.87	17.81	28.27	18.77	9.50	95
25	Lanzhou	NW	103.88	36.04	10/27/2014-12/3/2014	18.20	10.87	7.33	7.18	4.85	2.32	96
26	Panyu	PRD	113.35	23.00	11/7/2014-1/3/2015	20.35	7.91	12.55	19.19	7.34	11.84	97
27	Beijing	NCP	116.37	39.97	11/17/2014-12/13/2014	30.40	16.10	14.70	24.61	19.15	5.46	98
28	Beijing	NCP	116.37	39.97	12/1/2014-1/18/2015	31.80	18.40	14.30	20.61	16.59	4.02	99
29	Beijing	NCP	116.30	40.00	12/6/2014-2/27/2015	30.40	17.90	12.60	21.36	16.81	4.55	100
30	Shenzhen	PRD	113.90	22.60	12/31/2014-1/23/2015	18.45	7.93	10.51	15.78	5.43	10.35	101
31	Beijing	NCP	116.37	39.97	2/2/2015-4/1/2015	39.00	24.75	14.25	16.79	11.02	5.77	102
32	Mt. Wuzhi	OTR	109.49	18.84	3/18/2015-4/15/2015	4.90	0.00	4.89	5.03	1.21	3.82	103
33	Beijing	NCP	116.32	39.99	3/21/2015-5/20/2015	21.40	7.50	13.90	14.40	7.05	7.35	104
34	Mt. Yulong	OTR	100.20	27.20	3/22/2015-4/14/2015	3.88	0.50	3.37	7.91	2.71	5.20	105
35	Nanjing	YRD	118.73	32.01	4/13/2015-4/29/2015	12.69	5.64	7.04	20.01	7.90	12.11	106
36	Xiamen	OTR	118.05	24.60	5/1/2015-5/18/2015	13.07	3.61	9.46	7.01	2.79	4.22	107
37	Nam Co	OTR	90.98	30.77	5/30/2015-6/30/2015	0.71	0.13	0.59	0.57	0.10	0.47	108
38	Beijing	NCP	116.32	39.99	7/1/2015-8/19/2015	19.27	4.90	14.00	24.02	7.15	16.87	109
39	Shanghai	YRD	120.99	31.10	7/1/2015-8/31/2015	9.56	2.79	6.77	10.65	2.73	7.93	110
40	Beijing	NCP	116.32	39.99	8/15/2015-9/10/2015	13.80	3.90	9.80	16.84	5.81	11.03	111
41	Shanghai	YRD	120.99	31.10	9/1/2015-11/30/2015	13.23	3.79	9.43	11.07	3.40	7.67	110

No.	City	Region	Longitude	Latitude	Sampling Period	Observations [μg m-3] OA	Observations POA	Observations SOA	Model Simulations [μg m-3] OA	Model Simulations POA	Model Simulations SOA	Ref.
42	Beijing	NCP	116.37	39.97	9/4/2015-9/30/2015	18.31	6.33	11.97	19.07	7.74	11.33	112
43	Beijing	NCP	116.32	39.99	9/11/2015-10/10/2015	21.20	8.40	13.00	16.66	7.31	9.34	111
44	Beijing	NCP	116.37	39.97	10/1/2015-11/15/2015	25.00	12.50	12.50	21.23	11.92	9.31	83
45	Beijing	NCP	116.32	39.99	10/11/2015-12/4/2015	29.60	15.70	13.90	23.54	14.51	9.04	111
46	Nanjing	YRD	118.75	32.04	10/20/2015-11/19/2015	11.30	3.84	7.46	18.83	8.02	10.81	113
48	Beijing	NCP	116.37	39.97	12/1/2015-12/31/2015	48.80	30.26	18.54	35.65	24.58	11.07	114
47	Shanghai	YRD	120.99	31.10	12/1/2015-2/29/2016	17.80	4.76	13.04	18.18	7.78	10.39	110
49	Beijing	NCP	116.32	39.99	12/4/2015-2/6/2016	30.97	18.80	12.20	23.76	17.22	6.54	109
50	Handan	NCP	114.50	36.57	12/4/2015-2/5/2016	82.50	50.61	31.87	39.81	29.17	10.63	115
51	Nan'ao	OTR	117.02	23.42	12/22/2015-1/16/2016	7.06	2.61	4.45	13.41	4.38	9.04	116
52	Beijing	NCP	116.37	39.97	1/1/2016-1/31/2016	27.12	11.30	15.83	14.81	11.67	3.14	83
53	Shanghai	YRD	120.99	31.10	3/1/2016-5/31/2016	12.58	2.80	9.78	11.58	3.64	7.95	110
54	Dingri	OTR	86.95	28.36	4/12/2016-5/12/2016	2.39	1.04	1.34	6.42	2.53	3.89	117
55	Xingtai	NCP	114.37	37.18	4/30/2016-6/20/2016	11.59	2.54	9.04	13.37	4.88	8.49	118
56	Shanghai	YRD	120.99	31.10	6/1/2016-6/30/2016	10.97	2.65	8.32	9.35	2.28	7.07	110
57	Hangzhou	YRD	120.21	30.21	8/5/2016-8/21/2016	17.00	6.70	10.70	15.78	7.03	8.76	119
58	Shanghai	YRD	121.25	31.10	8/23/2016-9/10/2016	13.80	5.40	8.40	11.37	2.58	8.78	120
59	Beijing	NCP	116.31	39.99	9/3/2016-10/4/2016	21.80	6.10	15.70	20.16	7.41	12.74	121
60	Hangzhou	YRD	120.21	30.21	9/7/2016-9/23/2016	18.50	6.10	12.10	9.94	2.55	7.38	119
61	Beijing	NCP	116.37	39.97	10/1/2016-11/15/2016	15.03	5.26	9.77	17.45	9.75	7.71	83
62	Beijing	NCP	116.37	39.97	11/17/2016-12/13/2016	36.40	18.30	19.00	21.74	14.99	6.75	98
63	Shanghai	YRD	121.25	31.10	11/28/2016-1/13/2017	13.00	6.40	6.60	14.21	5.42	8.79	120
64	Gucheng	NCP	115.66	39.13	12/7/2016-1/8/2017	95.70	56.70	39.10	60.46	42.73	17.72	122
65	Beijing	NCP	116.37	39.97	12/17/2016-12/30/2016	53.70	22.60	30.90	36.40	24.74	11.65	123
66	Beijing	NCP	116.37	39.97	1/1/2017-1/31/2017	23.15	8.06	15.10	23.50	16.40	7.09	83
67	Shanghai	YRD	121.25	31.10	5/18/2017-6/4/2017	9.80	5.30	4.50	8.39	2.03	6.36	120
68	Beijing	NCP	116.31	39.99	5/23/2017-6/29/2017	12.02	3.49	8.53	13.03	4.06	8.97	121
69	Xinxiang	NCP	113.90	35.30	6/8/2017-6/25/2017	18.00	4.32	13.50	18.99	4.64	14.35	124
70	Waliguan	OTR	100.90	36.28	7/1/2017-7/31/2017	3.14	0.79	2.35	1.07	0.17	0.91	125
71	Xiamen	OTR	118.05	24.60	8/10/2017-9/10/2017	5.71	3.47	2.24	7.30	2.33	4.97	126
72	Beijing	NCP	116.37	39.97	10/1/2017-11/30/2017	11.60	4.60	7.00	14.41	7.90	6.50	127
73	Xianghe	NCP	116.96	39.80	10/3/2017-11/14/2017	13.90	7.92	5.98	21.52	11.58	9.94	128
74	Beijing	NCP	116.31	39.99	11/1/2017-12/18/2017	9.08	3.98	5.53	10.63	7.41	3.22	129
75	Hongkong	PRD	114.18	22.30	11/2/2017-12/13/2017	15.10	6.61	7.98	10.61	2.67	7.94	130
76	Xianghe	NCP	116.96	39.80	11/15/2017-2/1/2018	24.00	18.24	6.00	15.37	12.09	3.28	131
77	Guangzhou	PRD	113.37	23.15	11/20/2017-1/5/2018	17.30	5.36	12.11	17.20	5.42	11.78	132
78	Xiamen	OTR	118.05	24.60	12/3/2017-12/31/2017	9.94	2.49	7.44	13.43	5.02	8.41	133
79	Beijing	NCP	116.31	39.99	12/16/2017-1/10/2018	10.57	6.38	4.19	12.11	8.74	3.37	134
80	Beijing	NCP	116.37	39.97	12/1/2017-2/28/2018	9.10	4.10	5.00	11.89	8.79	3.11	127
81	Hongkong	PRD	114.18	22.30	12/24/2017-1/15/2018	6.80	2.35	4.45	11.01	3.07	7.94	135
82	Ningbo	YRD	121.90	29.75	1/23/2018-2/24/2018	3.81	0.56	3.25	8.93	3.06	5.87	136
83	Beijing	NCP	116.37	39.97	3/1/2018-5/31/2018	11.60	4.00	7.60	13.62	6.24	7.38	127
84	Xi'an	NW	109.02	34.34	5/19/2018-6/18/2018	6.81	1.51	5.30	9.15	3.96	5.20	137
85	Beijing	NCP	116.37	39.97	5/20/2018-6/23/2018	12.70	3.70	9.20	11.29	3.37	7.91	138
86	Beijing	NCP	116.37	39.97	6/1/2018-8/31/2018	11.60	4.00	7.60	15.78	3.93	11.85	127
87	Xiamen	OTR	118.05	24.60	8/1/2018-8/22/2018	7.89	1.60	6.30	7.56	1.90	5.66	133
88	Beijing	NCP	116.33	39.73	8/16/2018-9/16/2018	12.24	3.79	8.44	12.88	4.64	8.24	139
89	Rizhao	NCP	119.40	35.18	9/2/2018-9/29/2018	8.32	0.67	7.65	6.33	1.47	4.86	140
90	Guangzhou	PRD	113.36	23.14	10/1/2018-11/20/2018	14.70	5.30	9.70	16.86	4.60	12.26	141

No.	City	Region	Longitude	Latitude	Sampling Period	Observations [μg m-3]			Model Simulations [μg m-3]			Ref.
						OA	POA	SOA	OA	POA	SOA	
91	Beijing	NCP	116.37	39.97	10/15/2018-11/30/2018	20.50	5.90	14.60	15.36	8.57	6.79	142
92	Shanghai	YRD	121.43	31.17	10/31/2018-12/2/2018	9.44	4.80	4.65	11.55	3.00	8.54	143
93	Hongkong	PRD	114.25	22.21	11/1/2018-11/28/2018	5.99	0.81	5.18	7.92	1.48	6.43	144
94	Xinglong	NCP	117.67	40.40	11/10/2018-1/31/2019	5.40	2.20	3.20	7.41	4.17	3.24	145
95	Beijing	NCP	116.37	39.97	11/20/2018-12/25/2018	15.10	8.60	6.20	15.63	10.59	5.04	146
96	Xiamen	OTR	118.05	24.60	12/1/2018-12/22/2018	8.42	2.39	6.05	12.65	4.94	7.72	133
97	Xinxiang	NCP	113.92	35.28	12/1/2018-1/15/2019	22.58	10.29	12.28	21.77	12.67	9.10	147
98	Lanzhou	NW	104.13	35.95	12/4/2018-1/6/2019	10.50	5.00	5.50	5.29	2.48	2.81	148
99	Gucheng	NCP	115.73	39.15	12/8/2018-12/18/2018	42.00	33.18	8.82	23.40	18.99	4.42	149
100	Shenzhen	PRD	113.90	22.60	12/12/2018-1/2/2019	9.60	4.60	5.00	12.41	3.32	9.09	150
101	Beijing	NCP	116.30	39.94	10/14/2018-2/28/2019	19.06	11.30	7.76	14.46	9.37	5.09	151
102	Xi'an	NW	108.89	34.22	12/4/2018-3/15/2019	37.10	15.30	21.80	15.63	8.31	7.32	152
103	Sanmenxia	NCP	111.71	34.79	12/21/2018-1/21/2019	17.92	5.91	12.01	18.34	9.77	8.57	153
104	Beijing	NCP	116.05	40.00	1/18/2019-2/18/2019	11.30	5.20	6.10	10.47	7.82	2.65	154
105	Baoji	NW	107.14	34.35	2/26/2019-3/27/2019	16.02	6.41	9.61	6.98	2.77	4.21	155
106	Beijing	NCP	116.37	39.97	3/1/2019-4/15/2019	13.70	3.40	10.30	8.82	4.45	4.37	142
107	Rizhao	NCP	119.40	35.18	3/2/2019-3/29/2019	15.66	2.19	13.31	12.52	4.63	7.88	140
108	Xinglong	NCP	117.67	40.40	5/1/2019-5/31/2019	4.50	0.70	4.00	6.01	1.75	4.26	156
109	Xinglong	NCP	117.67	40.40	6/20/2019-7/26/2019	4.90	0.00	4.90	12.31	2.48	9.83	156
110	Xi'an	NW	108.89	34.22	6/22/2019-7/21/2019	14.00	4.40	9.60	7.17	2.03	5.14	157
111	Beijing	NCP	116.33	39.73	7/25/2019-8/21/2019	9.30	2.90	6.42	13.82	4.14	9.68	158
112	Handan	NCP	114.5	36.57	8/10/2019-9/17/2019	20.60	6.10	14.50	15.59	5.57	10.03	159
113	Lhasa	NW	91.03	29.65	8/31/2019-9/26/2019	3.88	2.56	1.32	0.35	0.11	0.23	160
114	Kaifeng	NCP	114.36	34.84	9/28/2019-11/1/2019	7.90	1.50	6.40	16.27	6.41	9.86	161
115	Xinglong	NCP	117.67	40.40	10/12/2019-11/12/2019	4.50	0.60	4.00	8.44	3.21	5.22	156
116	Shanghai	YRD	121.43	31.17	10/15/2019-12/31/2019	9.40	3.70	5.70	9.95	3.11	6.84	162
117	Xinglong	NCP	117.67	40.40	11/25/2019-12/25/2019	4.80	1.10	3.50	8.49	4.80	3.69	156
118	Gucheng	NCP	115.73	39.15	12/10/2019-1/13/2020	23.10	10.40	10.90	23.67	17.58	6.09	146
119	Qinling	NW	108.35	34.07	12/23/2019-2/7/2020	12.67	4.14	8.53	8.76	3.95	4.81	163
120	Beijing	NCP	116.37	39.97	1/1/2020-3/31/2020	16.61	2.18	14.34	15.10	8.90	6.20	164
121	Lanzhou	NW	103.86	36.05	1/14/2020-3/4/2020	12.41	6.16	6.24	4.27	2.55	1.72	165
122	Shenzhen	PRD	113.90	22.60	1/24/2020-2/24/2020	8.67	1.93	6.75	7.07	1.84	5.23	166
123	Laohukou	OTR	96.51	39.50	8/4/2020-8/29/2020	0.69	0.00	0.69	0.64	0.09	0.55	160
124	Xiamen	OTR	118.05	24.60	8/6/2020-8/31/2020	5.24	1.41	3.83	5.46	1.66	3.79	133
125	Beijing	NCP	116.37	39.97	11/1/2020-12/4/2020	9.40	5.00	4.40	12.02	7.25	4.77	167
126	Xiamen	OTR	118.05	24.60	12/1/2020-12/17/2020	6.28	1.80	4.48	8.91	2.84	6.07	133
Below are overlapped measurements that are used for observation validation												
127	Ziyang	OTR	104.64	30.15	12/3/2012-1/5/2013	21.50	6.20	15.40	--	--	--	168
128	Beijing	NCP	116.37	39.97	1/1/2013-2/1/2013	44.65	20.54	24.11	--	--	--	169
129	Xianghe	NCP	116.96	39.80	6/1/2013-6/30/2013	28.30	8.20	19.00	--	--	--	170
130	Xi'an	NW	108.88	34.23	12/13/2013-1/6/2014	127.50	100.32	21.00	--	--	--	89
131	Beijing	NCP	116.37	39.97	12/17/2013-1/17/2014	38.10	21.50	16.50	--	--	--	171
132	Beijing	NCP	116.37	39.97	1/1/2014-2/3/2014	27.27	8.45	18.82	--	--	--	172
133	Nanjing	YRD	118.71	32.04	8/11/2014-9/18/2014	6.10	3.00	3.10	--	--	--	173
134	Beijing	NCP	116.37	39.97	10/1/2014-10/27/2014	34.57	15.56	19.01	--	--	--	174
135	Beijing	NCP	116.37	39.97	10/14/2014-11/2/2014	29.40	7.50	14.90	--	--	--	175
136	Beijing	NCP	116.37	39.97	10/17/2014-11/2/2014	44.44	17.33	27.11	--	--	--	176
137	Beijing	NCP	116.37	39.97	11/13/2014-11/30/2014	38.10	23.80	13.10	--	--	--	99
138	Beijing	NCP	116.32	39.99	11/22/2014-3/20/2015	25.60	11.50	12.90	--	--	--	104

No.	City	Region	Longitude	Latitude	Sampling Period	Observations [μg m^{-3}]			Model Simulations [μg m^{-3}]			Ref.
						OA	POA	SOA	OA	POA	SOA	
139	Beijing	NCP	116.37	39.97	12/10/2014-12/31/2014	20.25	12.14	8.10	--	--	--	[174]
140	Beijing	NCP	116.38	40.00	12/29/2014-2/28/2015	38.38	26.86	11.90	--	--	--	[177]
141	Beijing	NCP	116.03	39.60	3/30/2015-4/30/2015	16.12	9.19	6.93	--	--	--	[95]
142	Beijing	NCP	116.32	39.99	5/21/2015-8/31/2015	13.40	3.20	6.60	--	--	--	[104]
143	Nam Co	OTR	90.95	30.77	5/31/2015-7/1/2015	1.36	0.00	1.36	--	--	--	[178]
144	Beijing	NCP	116.30	40.00	6/30/2015-7/27/2015	12.20	4.15	8.05	--	--	--	[124]
145	Beijing	NCP	116.32	39.99	9/1/2015-11/15/2015	18.90	8.90	10.90	--	--	--	[104]
146	Nanjing	PRD	118.75	32.04	10/20/2015-11/19/2015	25.20	7.06	18.14	--	--	--	[113]
147	Beijing	NCP	116.03	39.60	12/5/2015-1/7/2016	70.76	48.82	21.94	--	--	--	[95]
148	Beijing	NCP	116.37	39.97	5/25/2017-6/18/2017	10.42	3.02	7.41	--	--	--	[179]
149	Xiamen	OTR	118.05	24.60	8/1/2017-8/15/2017	9.64	2.82	6.81	--	--	--	[133]
150	Beijing	NCP	116.38	40.00	10/31/2017-12/5/2017	12.78	4.87	7.91	--	--	--	[180]
151	Beijing	NCP	116.30	40.00	12/11/2017-2/2/2018	11.90	6.10	5.80	--	--	--	[100]
152	Beijing	NCP	116.30	39.94	4/6/2018-7/2/2018	21.80	5.90	16.00	--	--	--	[181]
153	Beijing	NCP	116.37	39.97	5/20/2018-6/23/2018	12.70	3.70	9.10	--	--	--	[146]
154	Beijing	NCP	116.37	39.97	7/1/2018-8/5/2018	13.20	1.70	11.30	--	--	--	[142]
155	Beijing	NCP	116.37	39.97	10/1/2018-11/15/2018	10.79	3.92	6.87	--	--	--	[83]
156	Beijing	NCP	116.37	39.97	11/10/2018-1/31/2019	11.50	6.70	4.80	--	--	--	[145]
157	Xinglong	NCP	117.50	40.40	11/12/2018-12/24/2018	7.41	2.67	4.74	--	--	--	[182]
158	Beijing	NCP	116.37	39.97	1/1/2019-1/31/2019	13.56	6.54	7.02	--	--	--	[83]
159	Shanghai	YRD	121.43	31.17	10/15/2019-11/15/2019	8.80	2.93	5.87	--	--	--	[183]
160	Beijing	NCP	116.05	40.00	1/5/2020-2/7/2020	8.50	3.10	5.40	--	--	--	[154]
161	Yucheng	NW	111.05	35.04	11/1/2020-1/11/2021	24.50	9.20	15.30	--	--	--	[184]
162	Beijing	NCP	116.37	39.97	1/1/2021-3/31/2021	15.09	3.64	11.11	--	--	--	[164]

Table S2. Statistical values of NMB, RMSE, and *r* for the model-observation comparisons of the campaign-mean mass concentrations of OA, POA, and SOA in North China Plain (NCP), Yangzi River Delta (YRD), Pearl River Delta (PRD), and Northwest China (NW). "OBS" and "SIM" represent the observations and the simulations, respectively. The units of OBS, SIM, and RMSE are μg m^{-3}.

		NCP	YRD	PRD	NW
n		66	21	13	13
OA	OBS	23.05	15.45	13.54	15.08
	SIM	18.95	15.62	12.59	6.59
	NMB	-0.18	0.01	-0.07	-0.56
	RMSE	11.18	4.32	3.03	11.31
	r	0.91	0.84	0.85	0.85
POA	OBS	11.49	5.80	5.38	6.96
	SIM	11.32	6.55	3.97	3.27
	NMB	-0.01	0.13	-0.26	-0.53
	RMSE	6.48	2.76	2.73	5.67
	r	0.91	0.89	0.52	0.84
SOA	OBS	11.57	9.48	8.14	8.08
	SIM	7.63	9.07	8.62	3.32
	NMB	-0.34	-0.04	0.06	-0.59
	RMSE	6.92	3.52	2.03	6.05
	r	0.51	0.59	0.85	0.81

Table S3. Statistical values of NMB, RMSE, and *r* for the model-observation comparisons of the campaign-mean mass concentrations of OA, POA, and SOA in China from 2013 to 2020. "OBS" and "SIM" represent the observations and the simulations, respectively. The units of OBS, SIM, and RMSE are μg m^{-3}.

		2013	2014	2015	2016	2017	2018	2019	2020
n		16	12	22	15	14	21	18	8
OA	OBS	20.61	31.40	22.09	23.76	13.08	12.28	13.06	9.00
	SIM	18.20	20.54	17.64	18.43	13.40	12.32	11.63	7.78
	NMB	-0.12	-0.35	-0.20	-0.22	0.02	0.00	-0.11	-0.14
	RMSE	5.91	16.67	11.58	11.72	3.68	4.99	6.95	3.53
	r	0.86	0.79	0.86	0.97	0.80	0.83	0.57	0.73
POA	OBS	9.42	18.47	10.66	10.43	5.79	5.21	4.65	2.83
	SIM	9.99	13.57	9.20	9.77	6.46	5.49	5.46	3.64
	NMB	0.06	-0.27	-0.14	-0.06	0.12	0.05	0.17	0.28
	RMSE	4.34	10.86	5.81	4.21	3.62	3.66	3.43	2.83
	r	0.83	0.84	0.93	0.97	0.64	0.87	0.65	0.38
SOA	OBS	10.92	13.05	11.41	13.40	7.32	6.95	8.45	6.17
	SIM	8.21	6.97	8.45	8.66	6.94	6.83	6.16	4.14
	NMB	-0.25	-0.47	-0.26	-0.35	-0.05	-0.02	-0.27	-0.33
	RMSE	4.99	7.67	6.24	9.00	2.78	3.03	4.99	3.65
	r	0.63	0.31	0.44	0.70	0.72	0.36	0.41	0.61

Table S4. Emission factors and volatility distributions used in this study for IVOC.

Source Sector	Fuel	EF_{IVOC}/EF_{NMVOC}	Fractions for C^* bins			Reference
			10^6	10^5	10^{3-4}	
Residential	Biofuel	0.447	0.415	0.221	0.364	[23,24]
	Coal	0.510	0.135	0.255	0.611	[25,26]
	Other	0.255	0.606	0.181	0.212	[24]
Industry	Coal	0.120	0.155	0.262	0.584	[25,26]
	Other	0.178	0.338	0.309	0.353	[27]
Power	All	0.120	0.155	0.262	0.584	[25,26]
Transportation	Diesel	1.208	0.348	0.358	0.295	[28]
	Gasoline	0.044	0.649	0.207	0.143	[28-30]
Solvent	All	0.271	0.595	0.281	0.124	[31,32]
Open biomass burning	All	0.133	0.666	0.301	0.033	[33,34]
Ship	All	0.525[a]	0.158	0.236	0.606	[35]

[a] Using $EF_{IVOC}/EF_{POAinventory}$ instead because the correlation of IVOC with POA is much better than with NMVOC.

Table S5. Emission factors and volatility distributions used in this study for S/LVOC.

Source Sector	Fuel	$EF_{S/LVOC}/EF_{POAinventory}$	Fractions for C^* bins					Reference
			10^2	10^1	10^0	10^{-1}	10^{-2}	
Residential	Biofuel	1.0	0.445	0.268	0.110	0.140	0.037	[23,24]
	Coal		0.588	0.192	0.022	0.072	0.126	[25,26]
	Other		0.118	0.490	0.235	0.039	0.118	[24]
Industry	Coal		0.679	0.321	0.000	0.000	0.000	[25,26]
	Other		0.617	0.221	0.105	0.057	0.000	[27]
Power	All		0.679	0.321	0.000	0.000	0.000	[25,26]
Transportation	Diesel		0.327	0.483	0.059	0.045	0.086	[28]
	Gasoline		0.393	0.368	0.077	0.035	0.127	
Open biomass burning	All		0.333	0.167	0.167	0.000	0.333	[37]
Ship	All		0.108	0.087	0.078	0.082	0.646	[35]

Table S6. Model modifications to GEOS-Chem v13.3.1. The model simulations are evaluated against nationwide observations of OH and HONO from 2014 to 2019 as described in our previous study [3] as well as sulfate, nitrate, POA, and SOA concentrations obtained in 2017 (Table S1). Campaign-mean concentrations are used for the evaluations except that campaign-mean diurnal maximum is used for evaluating OH. The units of the RMSEs for OH, HONO, and PM components are 10^6 cm^{-3}, ppbv and μg m^{-3}, respectively.

Target Species	Model Parameter	Updated Parameterization	Performance Change
HONO<br>OH<br>Nitrate	γ_{HO2}[a]	0.08	Diurnal maximum OH[c]:<br>NMB: -0.73 → -0.25<br>RMSE: 2.28 → 1.34<br>(*n* = 4)
	HONO emissions from traffic[a]	1.7% of traffic NO_x emissions	
	HONO emissions from soil[a]	Scaling from soil NO_x emission with the considerations of biomes and soil water content	HONO:<br>NMB: -0.49 → -0.14<br>RMSE: 0.84 → 0.63<br>(*n* = 23)
	HONO emissions from open biomass burning[a]	Incorporating into GFED4s with emission factors from Andreae [47]	
	$\gamma_{NO2\text{-}a}$ on BC aerosol	10^{-5}	
	Heterogeneous oxidation of NO_2 on the ground[b]	Following the reaction suggested by Li, et al. [48] with land type-depend S_g/V and light intensity and RH-depend $\gamma_{NO2\text{-}g}$	Nitrate:<br>NMB: 0.43 → 0.39<br>RMSE: 4.37 → 4.76<br>(*n* = 14)
	Photolysis of nitrate	Following Kasibhatla, et al. [52] with a J_{scale} value of 50	
Sulfate	γ_{SO2}	10^{-6}	NMB: -0.40 → -0.20<br>RMSE: 3.20 → 2.46<br>(*n* = 14)
POA<br>SOA	Volatility bins of S/LVOC, POA, and SVOC-SOA[a]	Five volatility bins with C^* from 10^{-2} to 10^2 μg m^{-3}	POA:<br>NMB: 0.01 → 0.12<br>RMSE: 5.34 → 3.62<br>(*n* = 14)<br><br>SOA:<br>NMB: -0.38 → -0.05<br>RMSE: 3.81 → 2.78<br>(*n* = 14)
	Volatility distributions of S/LVOC emissions for individual sectors[b]	Based on the averages of laboratory combustion experiments for each sector	
	Volatility bins of IVOC[a]	Three volatility bins with C^* of 10^6, 10^5, and $10^{\leq 4}$	
	Fuel-based IVOC emissions	Scaling the corresponding NMVOC emissions by fuel-based EF_{IVOC}/EF_{NMVOC} from laboratory experiments for each sector	
	Volatility distributions of IVOC emissions for individual sectors[b]	Based on the averages of laboratory experiments for each sector	
	SOA yields of IVOC under high NO_x condition[a]	SOA yields of the photooxidation of C_{12}–C_{14}, C_{15}–C_{16}, and C_{17} *n*-alkanes are used for three IVOC bins with corrections for vapor wall losses (Table S7)	
	SOA yields of BVOC and AVOC	Fitting from laboratory results with corrections for vapor wall losses (Table S7)	

[a]: Model updates that are consistent with our previous study using GEOS-Chem 12.6.3 [3].

[b]: Model modifications that are firstly implemented in our previous study and further updated in this study.

[c]: Only evaluations for wintertime measurements (removing overlapped ones) are presented here because the simulated concentrations in the default and revised versions are similar in other three seasons.

Table S7. Updated SOA yield parameterizations for various precursors at 298 K under low and high NO_x conditions and the correction factors to account for the vapor wall losses [56]. The saturation concentration (C^*) is in unit of μg m^{-3}. The five-bin S/LVOC partitions directly to the particle phase after emission to form POA. The remaining gaseous S/LVOC is assumed to be oxidized by OH at a rate constant of 2×10^{-11} cm^3 $molec^{-1}$ s^{-1} to form condensable products with 100 times lower volatilities that partition to the particle phase to form SOA.

Lumped Precursors	Oxidant (high NO_x or low NO_x)	mass-based stoichiometric coefficients *(α)* for C^*					SOA yield at 10 μg m^{-3}	Correction factors to account for vapor wall losses	Ref.
		Non-volatile	0.1	1	10	100			
Limonene	OH, O_3 (NO)	-	0.000	0.474	0.117	1.419	0.62	1.0	[231]
	OH, O_3 (HO_2)	-	0.000	0.366	0.321	0.817	0.57		
Monnoterpenes except limonene	OH, O_3 (NO)	-	0.027	0.000	0.000	0.531	0.08	1.0	[232-235]
	OH, O_3 (HO_2)	-	0.139	0.000	0.077	0.677	0.24		[236-238]
Sesquiterpenes	OH, O_3 (NO)	-	0.000	0.000	0.723	0.725	0.43	1.0	[239]
	OH, O_3 (HO_2)	-	0.079	0.000	0.373	0.661	0.32		[240]
Isoprene	OH (NO)	-	0.000	0.000	0.000	0.017	0.00	2.2	[241]
Benzene	OH (NO)	-	0.048	0.024	0.000	0.835	0.15	1.2	[238,242-247]
	OH (HO_2)	-	0.000	0.000	0.000	0.485	0.04	1.9	
Toluene	OH (NO)	-	0.023	0.000	0.105	0.084	0.08	1.2	[238,242-247]
	OH (HO_2)	-	0.000	0.000	0.000	0.959	0.09	1.9	
Xylene	OH (NO)	-	0.016	0.000	0.049	0.076	0.05	1.2	[238,242-247]
	OH (HO_2)	-	0.006	0.000	0.000	0.549	0.06	1.9	
IVOC6	OH (NO)	-	0.011	0.052	0.201	0.296	0.19	1.2	[248]
IVOC5		-	0.049	0.078	0.439	0.271	0.36		
IVOC4		-	0.063	0.089	0.550	0.200	0.44		
All IVOC	OH (HO_2)	0.730	-	-	-	-	0.73		[249]

Table S8. Descriptions of the simulation scenarios.

Simulation Runs	Simulation Year (*i*)	Control Year on Various Factors				
		Meteorological fields	Emissions of OA precursors	Emissions of SO_2	Emissions of NO_x	All Other Emissions
Base	2013-2020	--	--	--	--	--
Fix_ALLEMIS	2014-2020	--	2013	2013	2013	2013
Fix_MET	2014-2020	2013	--	--	--	--
CTL_ALLEMIS	2017	--	2013	2013	2013	2013
	2020	--	2017	2017	2017	2017
CTL_MET	2017	2013	--	--	--	--
	2020	2017	--	--	--	--
CTL_OAP	2017	--	2013	--	--	--
	2020	--	2017	--	--	--
CTL_OTR	2017	--	--	2013	2013	2013
	2020	--	--	2017	2017	2017
CTL_OTR_woSO_2	2017	--	--	--	2013	2013
	2020	--	--	--	2017	2017
CTL_OTR_woNO_x	2017	--	--	2013	--	2013
	2020	--	--	2017	--	2017
Half_ResIVOC	2019	--	50% of residential IVOC	--	--	--
Half_ResS/LVOC	2019	--	50% of residential S/LVOC	--	--	--
Half_ResALL	2019	--	50% of all residential OA precursors	--	--	--
Zero_ResALL	2019	--	0% of all residential OA precursors	--	--	--
Half_SolIVOC	2019	--	50% of solvent use IVOC	--	--	--
Half_SolVOC	2019	--	50% of solvent use VOC	--	--	--
Half_SolALL	2019	--	50% of all solvent-use OA precursors	--	--	--
Half_IndIVOC	2019	--	50% of industry IVOC	--	--	--
Half_IndS/LVOC	2019	--	50% of industry S/LVOC	--	--	--
Half_IndALL	2019	--	50% of all industry OA precursors	--	--	--

Note:

1. The contributions of meteorological variations and emission controls of OA precursors, other pollutants (non-OA-precursors), SO_2, and NO_x to the POA or SOA concentration variations (ΔC) during the two action periods are estimated by $Base_i$ - CTL_MET_i, $Base_i$ - CTL_OAP_i , $Base_i$ - CTL_OTR_i, $CTL\_OTR\_woSO_{2\,i}$ - CTL_OTR_i and $CTL\_OTR\ \_woNO_{x\,i}$- CTL_OTR_i, respectively, for which *i* is the simulation year of 2017 for the first-action period and 2020 for the second-action period. The calculation of CTL_OTR_i - $CTL\_ALLEMIS_i$ may also represent the ΔC led by emission controls of OA precursors, the base pollution level of which is higher than the calculation of $Base_i$ - CTL_OAP_i. We obtain similar results for the two calculations, suggesting negligible nonlinear effects of the base-year pollution level on the calculations.
2. The yearly variations of concentrations under fixed 2013-emission and 2013-meteorology scenarios are calculated by $Fix\_ALLEMIS_i$ / $Base_{2013}$ -1 and Fix_MET_i / $Base_{2013}$ -1, respectively, for which *i* is the simulation year from 2014 to 2020.
3. The reductions of population-weighted OA in different policy cases that cut off precursor emissions in different sectors are calculated by the differences between $Base_{2019}$ and corresponding cases (Half_ and Zero_ series).

Table S9. Global burdens of OA under different model schemes.

Source	Category	Precursor	Global burden [Tg]				
			Simple scheme [Pai et al., 2020]	Complex scheme [Pye et al., 2010][a]	Hodzic scheme[b] [Hodzic et al., 2016]	Complex scheme [Pye et al., 2010]	Revised complex scheme [This study]
			Non-volatile POA treatment			Semivolatile POA treatment	
			OM:OC=1.4 for EPOA and 2.1 for OPOA[c]			two-product surrogates	5-bin S/LVOC volatility distributions
			2013	2000	2005-2008 mean	2000	2013[d]
Anthropogenic and biomass burning	**POA**		**0.84**	**0.92**	**0.94**	**0.03**	**0.47**
		EPOA	0.06	-- [e]	--	0.03	0.47
		OPOA	0.78	--	--	Not applicable	Not applicable
	**SOA**		**0.69**	--	**0.29**	**(~1.0)**[f]	**1.30**
		S/LVOC	--	Not included	0.21	0.81[g]	0.77[g]
		IVOC	--			0.09	0.48
		Aromatics	--	--	0.08	Not specified	0.05
	**$OA_{anth/bb}$**		**1.53**	**--**	**1.23**	**(~1.0)**[f]	**1.77**
All except marine	**OA**		**1.86**	**1.64**	**1.82**	**1.65**	**2.15**
Model results are evaluated by			Aircraft OA data		IMPROVE (US) and EMEP (Europe) OC data; AMS OOA data for rural/background; Aircraft OA data		NCP and YRD OC data in China; AMS PMF (POA and OOA) data; Precursor concentrations and volatility distributions

[a] Pai et al. [2020] also reported 2013 burden for complex scheme. Their burden values are greater than those reported by Pye et al. [2010] when the primary OC emissions in the two studies are similar (31.2 vs 29.0 Tg C/yr). We list only the values from Pye et al. [2010] in the table.

[b] The ND_DPH setting is considered as the Hodzic scheme used by Brew et al. [2023]. Brew et al. [2023] further improved aromatic SOA parameterization.

[c] The OM:OC ratio is 2.1 for both EPOA and OPOA in [Pye et al., 2010] while the OM:OC ratios are not reported in [Hodzic et al., 2016].

[d] The burden is calculated on the default configuration of GEOS-Chem (v13.3.1) with the revised complex scheme for OA. To be consistent with other studies, the wet deposition of major inorganic aerosols and their precursors were not updated with the parameterization introduced by Luo et al. (9, 10) in the burden calculation, while all the other simulations have this update as described in Sect. S1 (Model configurations).

[e] Included in the calculation but the values are not specified in the literature.

[f] The value is not specified in the literature. The aromatic SOA burden should be less than 0.10 Tg.

[g] Labeled as the OPOA tracer.

Supplementary References

1 Bey, I. *et al.* Global modeling of tropospheric chemistry with assimilated meteorology: Model description and evaluation. *J. Geophys. Res. Atmos.* **106**, 23073-23095, doi:10.1029/2001JD000807 (2001).

2 Pye, H. O. T. *et al.* Effect of changes in climate and emissions on future sulfate-nitrate-ammonium aerosol levels in the United States. *J. Geophys. Res. Atmos.* **114**, doi:10.1029/2008JD010701 (2009).

3 Miao, R. *et al.* Process-based and observation-constrained SOA simulations in China: the role of semivolatile and intermediate-volatility organic compounds and OH levels. *Atmos. Chem. Phys.* **21**, 16183-16201, doi:10.5194/acp-21-16183-2021 (2021).

4 Lin, S. J. & Rood, R. B. Multidimensional flux-form semi-Lagrangian transport schemes. *Mon. Weather Rev.* **124**, 2046-2070, doi:Doi 10.1175/1520-0493(1996)124<2046:Mffslt>2.0.Co;2 (1996).

5 Lin, J. T. & McElroy, M. B. Impacts of boundary layer mixing on pollutant vertical profiles in the lower troposphere: Implications to satellite remote sensing. *Atmos. Environ.* **44**, 1726-1739, doi:10.1016/j.atmosenv.2010.02.009 (2010).

6 Wesely, M. L. Parameterization of Surface Resistances to Gaseous Dry Deposition in Regional-Scale Numerical-Models. *Atmos. Environ.* **23**, 1293-1304, doi:10.1016/0004-6981(89)90153-4 (1989).

7 Zhang, L. M., Gong, S. L., Padro, J. & Barrie, L. A size-segregated particle dry deposition scheme for an atmospheric aerosol module. *Atmos. Environ.* **35**, 549-560, doi:10.1016/S1352-2310(00)00326-5 (2001).

8 Jaegle, L. *et al.* Nitrogen Oxides Emissions, Chemistry, Deposition, and Export Over the Northeast United States During the WINTER Aircraft Campaign. *J. Geophys. Res. Atmos.* **123**, 12368-12393, doi:10.1029/2018JD029133 (2018).

9 Liu, H., Jacob, D. J., Bey, I. & Yantosca, R. M. Constraints from ^{210}Pb and ^{7}Be on wet deposition and transport in a global three-dimensional chemical tracer model driven by assimilated meteorological fields. *J. Geophys. Res. Atmos.* **106**, 12109-12128, doi:10.1029/2000JD900839 (2001).

10 Luo, G., Yu, F. & Moch, J. M. Further improvement of wet process treatments in GEOS-Chem v12.6.0: impact on global distributions of aerosols and aerosol precursors. *Geosci. Model Dev.* **13**, 2879-2903, doi:10.5194/gmd-13-2879-2020 (2020).

11 Hoesly, R. M. *et al.* Historical (1750-2014) anthropogenic emissions of reactive gases and aerosols from the Community Emissions Data System (CEDS). *Geosci. Model Dev.* **11**, 369-408, doi:10.5194/gmd-11-369-2018 (2018).

12 Guenther, A. B. *et al.* The Model of Emissions of Gases and Aerosols from Nature version 2.1 (MEGAN2.1): an extended and updated framework for modeling biogenic emissions. *Geosci. Model Dev.* **5**, 1471-1492, doi:10.5194/gmd-5-1471-2012 (2012).

13 van der Werf, G. R. *et al.* Global fire emissions estimates during 1997–2016. *Earth Syst. Sci. Data* **9**, 697-720, doi:10.5194/essd-9-697-2017 (2017).

14 Murray, L. T., Jacob, D. J., Logan, J. A., Hudman, R. C. & Koshak, W. J. Optimized regional and interannual variability of lightning in a global chemical transport model constrained by LIS/OTD satellite data. *J. Geophys. Res. Atmos.* **117**, 14, doi:10.1029/2012JD017934 (2012).

15 Hudman, R. C. *et al.* Steps towards a mechanistic model of global soil nitric oxide emissions: implementation and space based-constraints. *Atmos. Chem. Phys.* **12**, 7779-7795, doi:10.5194/acp-12-7779-2012 (2012).

16 Chen, Y. *et al.* Interannual variation of reactive nitrogen emissions and their impacts on $PM_{2.5}$ air pollution in China during 2005-2015. *Environ. Res. Lett.* **16**, 125004, doi:10.1088/1748-9326/ac3695 (2021).
17 Zheng, B. *et al.* Trends in China's anthropogenic emissions since 2010 as the consequence of clean air actions. *Atmos. Chem. Phys.* **18**, 14095-14111, doi:10.5194/acp-18-14095-2018 (2018).
18 Li, M. *et al.* Persistent growth of anthropogenic non-methane volatile organic compound (NMVOC) emissions in China during 1990–2017: drivers, speciation and ozone formation potential. *Atmos. Chem. Phys.* **19**, 8897-8913, doi:10.5194/acp-19-8897-2019 (2019).
19 Liu, Y. *et al.* Drivers of Increasing Ozone during the Two Phases of Clean Air Actions in China 2013–2020. *Environ. Sci. Technol.*, doi:10.1021/acs.est.3c00054 (2023).
20 Li, M. *et al.* Mapping Asian anthropogenic emissions of non-methane volatile organic compounds to multiple chemical mechanisms. *Atmos. Chem. Phys.* **14**, 5617-5638, doi:10.5194/acp-14-5617-2014 (2014).
21 Zhao, Y. *et al.* Intermediate Volatility Organic Compound Emissions from On-Road Diesel Vehicles: Chemical Composition, Emission Factors, and Estimated Secondary Organic Aerosol Production. *Environ Sci Technol* **49**, 11516-11526, doi:10.1021/acs.est.5b02841 (2015).
22 Zhao, Y. *et al.* Intermediate Volatility Organic Compound Emissions from On-Road Gasoline Vehicles and Small Off-Road Gasoline Engines. *Environ Sci Technol* **50**, 4554-4563, doi:10.1021/acs.est.5b06247 (2016).
23 Huang, G. *et al.* Emission factors and chemical profile of I/SVOCs emitted from household biomass stove in China. *Sci. Total Environ.* **842**, 156940, doi:10.1016/j.scitotenv.2022.156940 (2022).
24 Stewart, G. J. *et al.* Comprehensive organic emission profiles, secondary organic aerosol production potential, and OH reactivity of domestic fuel combustion in Delhi, India. *Environ. Sci.: Atmos.* **1**, 104-117, doi:10.1039/d0ea00009d (2021).
25 Cai, S. *et al.* Time-Resolved Intermediate-Volatility and Semivolatile Organic Compound Emissions from Household Coal Combustion in Northern China. *Environ. Sci. Technol.* **53**, 9269-9278, doi:10.1021/acs.est.9b00734 (2019).
26 Chang, X. *et al.* Full-volatility emission framework corrects missing and underestimated secondary organic aerosol sources. *One Earth* **5**, 403-412, doi:10.1016/j.oneear.2022.03.015 (2022).
27 Qi, L. *et al.* Intermediate-Volatility Organic Compound Emissions from Nonroad Construction Machinery under Different Operation Modes. *Environ Sci Technol* **53**, 13832-13840, doi:10.1021/acs.est.9b01316 (2019).
28 Lu, Q., Zhao, Y. & Robinson, A. L. Comprehensive organic emission profiles for gasoline, diesel, and gas-turbine engines including intermediate and semi-volatile organic compound emissions. *Atmos. Chem. Phys.* **18**, 17637-17654, doi:10.5194/acp-18-17637-2018 (2018).
29 Tang, R. *et al.* Measurement report: Distinct emissions and volatility distribution of intermediate-volatility organic compounds from on-road Chinese gasoline vehicles: implication of high secondary organic aerosol formation potential. *Atmos. Chem. Phys.* **21**, 2569-2583, doi:10.5194/acp-21-2569-2021 (2021).
30 Liu, Y. *et al.* Identification of two main origins of intermediate-volatility organic compound emissions from vehicles in China through two-phase simultaneous characterization. *Environ Pollut* **281**, 117020, doi:10.1016/j.envpol.2021.117020 (2021).

31 Seltzer, K. M. *et al.* Reactive organic carbon emissions from volatile chemical products. *Atmos. Chem. Phys.* **21**, 5079-5100, doi:10.5194/acp-21-5079-2021 (2021).
32 Tanzer-Gruener, R. *et al.* Watching Paint Dry: Organic Vapor Emissions from Architectural Coatings and their Impact on Secondary Organic Aerosol Formation. *Environ. Sci. Technol.* **56**, 11236-11245, doi:10.1021/acs.est.2c02478 (2022).
33 Koss, A. R. *et al.* Non-methane organic gas emissions from biomass burning: identification, quantification, and emission factors from PTR-ToF during the FIREX 2016 laboratory experiment. *Atmos. Chem. Phys.* **18**, 3299-3319, doi:10.5194/acp-18-3299-2018 (2018).
34 Hatch, L. E. *et al.* Multi-instrument comparison and compilation of non-methane organic gas emissions from biomass burning and implications for smoke-derived secondary organic aerosol precursors. *Atmos. Chem. Phys.* **17**, 1471-1489, doi:10.5194/acp-17-1471-2017 (2017).
35 Huang, C. *et al.* Intermediate Volatility Organic Compound Emissions from a Large Cargo Vessel Operated under Real-World Conditions. *Environ Sci Technol* **52**, 12934-12942, doi:10.1021/acs.est.8b04418 (2018).
36 Turpin, B. J. & Lim, H.-J. Species Contributions to PM2.5 Mass Concentrations: Revisiting Common Assumptions for Estimating Organic Mass. *Aerosol Sci. Technol.* **35**, 602-610, doi:10.1080/02786820119445 (2001).
37 May, A. A. *et al.* Gas-particle partitioning of primary organic aerosol emissions: 3. Biomass burning. *J. Geophys. Res. Atmos.* **118**, 11327-11338, doi:10.1002/jgrd.50828 (2013).
38 Li, M. *et al.* Anthropogenic emission inventories in China: a review. *Natl. Sci. Rev.* **4**, 834-866, doi:10.1093/nsr/nwx150 (2017).
39 Alexander, B., Park, R. J., Jacob, D. J. & Gong, S. L. Transition metal-catalyzed oxidation of atmospheric sulfur: Global implications for the sulfur budget. *J. Geophys. Res. Atmos.* **114**, doi:10.1029/2008JD0486 (2009).
40 Chen, Q. *et al.* Sulfate production by reactive bromine: Implications for the global sulfur and reactive bromine budgets. *Geophys. Res. Lett.* **44**, 7069-7078, doi:10.1002/2017gl073812 (2017).
41 Wang, Y. *et al.* Enhanced sulfate formation during China's severe winter haze episode in January 2013 missing from current models. *J. Geophys. Res. Atmos.* **119**, 10425-10440, doi:10.1002/2013JD021426 (2014).
42 Moch, J. M. *et al.* Global Importance of Hydroxymethanesulfonate in Ambient Particulate Matter: Implications for Air Quality. *J Geophys Res Atmos* **125**, e2020JD032706, doi:10.1029/2020JD032706 (2020).
43 Holmes, C. D. *et al.* The Role of Clouds in the Tropospheric NO_x Cycle: A New Modeling Approach for Cloud Chemistry and Its Global Implications. *Geophys. Res. Lett.* **46**, 4980-4990, doi:10.1029/2019GL081990 (2019).
44 Yu, C. *et al.* Measurement of heterogeneous uptake of NO2 on inorganic particles, sea water and urban grime. *J Environ Sci (China)* **106**, 124-135, doi:10.1016/j.jes.2021.01.018 (2021).
45 Rappengluck, B. *et al.* Radical precursors and related species from traffic as observed and modeled at an urban highway junction. *J. Air Waste Manage. Assoc.* **63**, 1270-1286, doi:10.1080/10962247.2013.822438 (2013).
46 Oswald, R. *et al.* HONO Emissions from Soil Bacteria as a Major Source of Atmospheric Reactive Nitrogen. *Science* **341**, 1233-1235, doi:10.1126/science.1242266 (2013).

47 Andreae, M. O. Emission of trace gases and aerosols from biomass burning – an updated assessment. *Atmos. Chem. Phys.* **19**, 8523-8546, doi:10.5194/acp-19-8523-2019 (2019).
48 Li, G. *et al.* Impacts of HONO sources on the photochemistry in Mexico City during the MCMA-2006/MILAGO Campaign. *Atmos. Chem. Phys.* **10**, 6551-6567, doi:10.5194/acp-10-6551-2010 (2010).
49 Vogel, B., Vogel, H., Kleffmann, J. & Kurtenbach, R. Measured and simulated vertical profiles of nitrous acid - Part II. Model simulations and indications for a photolytic source. *Atmos. Environ.* **37**, 2957-2966, doi:10.1016/S1352-2310(03)00243-7 (2003).
50 Sarwar, G. *et al.* A comparison of CMAQ HONO predictions with observations from the northeast oxidant and particle study. *Atmos. Environ.* **42**, 5760-5770, doi:10.1016/j.atmosenv.2007.12.065 (2008).
51 Liu, J. *et al.* Light-Enhanced Heterogeneous Conversion of NO_2 to HONO on Solid Films Consisting of Fluorene and Fluorene/Na_2SO_4: An Impact on Urban and Indoor Atmosphere. *Environ. Sci. Technol.* **54**, 11079-11086, doi:10.1021/acs.est.0c02627 (2020).
52 Kasibhatla, P. *et al.* Global impact of nitrate photolysis in sea-salt aerosol on NO_x, OH, and O_3 in the marine boundary layer. *Atmos. Chem. Phys.* **18**, 11185-11203, doi:10.5194/acp-18-11185-2018 (2018).
53 Tan, Z. *et al.* No Evidence for a Significant Impact of Heterogeneous Chemistry on Radical Concentrations in the North China Plain in Summer 2014. *Environ. Sci. Technol.* **54**, 5973-5979, doi:10.1021/acs.est.0c00525 (2020).
54 Robinson, A. L. *et al.* Rethinking Organic Aerosols: Semivolatile Emissions and Photochemical Aging. *Science* **315**, 1259-1262, doi:10.1126/science.1133061 (2007).
55 Pye, H. O. T., Chan, A. W. H., Barkley, M. P. & Seinfeld, J. H. Global modeling of organic aerosol: the importance of reactive nitrogen (NO_x and NO_3). *Atmos. Chem. Phys.* **10**, 11261-11276, doi:10.5194/acp-10-11261-2010 (2010).
56 Zhang, X. *et al.* Influence of vapor wall loss in laboratory chambers on yields of secondary organic aerosol. *Proc. Natl. Acad. Sci. U. S. A.* **111**, 5802-5807, doi:10.1073/pnas.1404727111 (2014).
57 Marais, E. A. *et al.* Aqueous-phase mechanism for secondary organic aerosol formation from isoprene: application to the southeast United States and co-benefit of SO_2 emission controls. *Atmos. Chem. Phys.* **16**, 1603-1618, doi:10.5194/acp-16-1603-2016 (2016).
58 Fisher, J. A. *et al.* Organic nitrate chemistry and its implications for nitrogen budgets in an isoprene- and monoterpene-rich atmosphere: constraints from aircraft ($SEAC^4RS$) and ground-based (SOAS) observations in the Southeast US. *Atmos. Chem. Phys.* **16**, 5969-5991, doi:10.5194/acp-16-5969-2016 (2016).
59 Pai, S. J. *et al.* An evaluation of global organic aerosol schemes using airborne observations. *Atmos. Chem. Phys.* **20**, 2637-2665, doi:10.5194/acp-20-2637-2020 (2020).
60 Sareen, N., Waxman, E. M., Turpin, B. J., Volkamer, R. & Carlton, A. G. Potential of Aerosol Liquid Water to Facilitate Organic Aerosol Formation: Assessing Knowledge Gaps about Precursors and Partitioning. *Environ. Sci. Technol.* **51**, 3327-3335, doi:10.1021/acs.est.6b04540 (2017).
61 Pye, H. O. T. & Seinfeld, J. H. A global perspective on aerosol from low-volatility organic compounds. *Atmos. Chem. Phys.* **10**, 4377-4401, doi:10.5194/acp-10-4377-2010 (2010).
62 Breiman, L. Random forests. *Mach. Learn.* **45**, 5-32, doi: 10.1023/A:1010933404324 (2001).

63 Lundberg, S. M. & Lee, S. I. A Unified Approach to Interpreting Model Predictions. *Adv. Neural Inf. Process.* **30** (2017).
64 Ding, F., Zhu, Z., Lu, X., Yang, L. & Zhang, L. Characteristics analysis of carbon components in $PM_{2.5}$ in Nanjing from 2014 to 2018. *Enviromental Monitoring in China* **36**, 165-172 (2020).
65 Xie, M. *et al.* Evaluating the influence of constant source profile presumption on PMF analysis of $PM_{2.5}$ by comparing long- and short-term hourly observation-based modeling. *Environ. Pollut.* **314**, 120273, doi:10.1016/j.envpol.2022.120273 (2022).
66 Wang, M. *et al.* Measurement report: Characterisation and sources of the secondary organic carbon in a Chinese megacity over 5 years from 2016 to 2020. *Atmos. Chem. Phys.* **22**, 12789-12802, doi:10.5194/acp-22-12789-2022 (2022).
67 Ji, D. *et al.* Impact of air pollution control measures and regional transport on carbonaceous aerosols in fine particulate matter in urban Beijing, China: insights gained from long-term measurement. *Atmos. Chem. Phys.* **19**, 8569-8590, doi:10.5194/acp-19-8569-2019 (2019).
68 Zhao, L. *et al.* Changes of chemical composition and source apportionment of $PM_{2.5}$ during 2013-2017 in urban Handan, China. *Atmos. Environ.* **206**, 119-131, doi:10.1016/j.atmosenv.2019.02.034 (2019).
69 Ji, D. *et al.* The carbonaceous aerosol levels still remain a challenge in the Beijing-Tianjin-Hebei region of China: Insights from continuous high temporal resolution measurements in multiple cities. *Environ. Int.* **126**, 171-183, doi:10.1016/j.envint.2019.02.034 (2019).
70 Peng, M. *et al.* Seasonal characteristics and sources of $PM_{2.5}$ in Tangshan. *Ecology and Environmental Sciences* **29**, 1855-1861, doi:10.16258/j.cnki.1674-5906.2020.09.018 (2020).
71 Dai, L., Zhang, L., Chen, D. & Zhao, Y. Assessment of carbonaceous aerosols in suburban Nanjing under air pollution control measures: Insights from long-term measurements. *Environ. Res.* **212**, 113302, doi:10.1016/j.envres.2022.113302 (2022).
72 Chang, Y. *et al.* Assessment of carbonaceous aerosols in Shanghai, China - Part 1: long-term evolution, seasonal variations, and meteorological effects. *Atmos. Chem. Phys.* **17**, 9945-9964, doi:10.5194/acp-17-9945-2017 (2017).
73 Xu, J. *et al.* Insights into the characteristics and sources of primary and secondary organic carbon: High time resolution observation in urban Shanghai. *Environ. Pollut.* **233**, 1177-1187, doi:10.1016/j.envpol.2017.10.003 (2018).
74 Liu, Z. *et al.* Characteristics of $PM_{2.5}$ mass concentrations and chemical species in urban and background areas of China: emerging results from the CARE-China network. *Atmos. Chem. Phys.* **18**, 8849-8871, doi:10.5194/acp-18-8849-2018 (2018).
75 Chow, J. C. Measurement Methods to Determine Compliance with Ambient Air Quality Standards for Suspended Particles. *J. Air Waste Manage. Assoc.* **45**, 320-382, doi:10.1080/10473289.1995.10467369 (1995).
76 Hu, W. *et al.* Seasonal variations in high time-resolved chemical compositions, sources, and evolution of atmospheric submicron aerosols in the megacity Beijing. *Atmos. Chem. Phys.* **17**, 9979-10000, doi:10.5194/acp-17-9979-2017 (2017).
77 Tang, L. *et al.* Components and optical properties of submicron aerosol during the lasting haze period in Nanjing. *Chin. Sci. Bull.* **59**, 1955, doi:10.1360/972013-1098 (2014).
78 Lee, B. P., Li, Y. J., Yu, J. Z., Louie, P. K. K. & Chan, C. K. Characteristics of submicron particulate matter at the urban roadside in downtown Hong Kong-Overview of 4 months

of continuous high-resolution aerosol mass spectrometer measurements. *J. Geophys. Res. Atmos.* **120**, 7040-7058, doi:10.1002/2015JD023311 (2015).
79 Zhang, Y. *et al.* Insights into characteristics, sources, and evolution of submicron aerosols during harvest seasons in the Yangtze River delta region, China. *Atmos. Chem. Phys.* **15**, 1331-1349, doi:10.5194/acp-15-1331-2015 (2015).
80 Zhu, Q. *et al.* Characterization of Organic Aerosol at a Rural Site in the North China Plain Region: Sources, Volatility and Organonitrates. *Adv. Atmos. Sci.* **38**, 1115-1127, doi:10.1007/s00376-020-0127-2 (2021).
81 Zhang, Y. J. *et al.* Aging of atmospheric organic aerosol during summertime in Nanjing: insights from on-line measurement. *Sci. Sin. Chim.* **44**, 1654, doi:10.1360/n032013-00049 (2014).
82 Du, W. *et al.* Chemical characterization of submicron aerosol and particle growth events at a national background site (3295 m a.s.l.) on the Tibetan Plateau. *Atmos. Chem. Phys.* **15**, 10811-10824, doi:10.5194/acp-15-10811-2015 (2015).
83 Li, J. *et al.* Significant changes in autumn and winter aerosol composition and sources in Beijing from 2012 to 2018: Effects of clean air actions. *Environ. Pollut.* **268**, 115855, doi:10.1016/j.envpol.2020.115855 (2021).
84 Zhu, Q. *et al.* Improved source apportionment of organic aerosols in complex urban air pollution using the multilinear engine (ME-2). *Atmos. Meas. Tech.* **11**, 1049-1060, doi:10.5194/amt-11-1049-2018 (2018).
85 Sun, C. *et al.* Continuous measurements at the urban roadside in an Asian megacity by Aerosol Chemical Speciation Monitor (ACSM): particulate matter characteristics during fall and winter seasons in Hong Kong. *Atmos. Chem. Phys.* **16**, 1713-1728, doi:10.5194/acp-16-1713-2016 (2016).
86 Zhang, Y. *et al.* Significant concentration changes of chemical components of PM_1 in the Yangtze River Delta area of China and the implications for the formation mechanism of heavy haze-fog pollution. *Sci. Total Environ.* **538**, 7-15, doi:10.1016/j.scitotenv.2015.06.104 (2015).
87 Zhang, Y. *et al.* Chemical composition, sources and evolution processes of aerosol at an urban site in Yangtze River Delta, China during wintertime. *Atmos. Environ.* **123**, 339-349, doi:10.1016/j.atmosenv.2015.08.017 (2015).
88 Sun, Y. *et al.* Real-Time Characterization of Aerosol Particle Composition above the Urban Canopy in Beijing: Insights into the Interactions between the Atmospheric Boundary Layer and Aerosol Chemistry. *Environ. Sci. Technol.* **49**, 11340-11347, doi:10.1021/acs.est.5b02373 (2015).
89 Elser, M. *et al.* New insights into $PM_{2.5}$ chemical composition and sources in two major cities in China during extreme haze events using aerosol mass spectrometry. *Atmos. Chem. Phys.* **16**, 3207-3225, doi:10.5194/acp-16-3207-2016 (2016).
90 Xu, J. *et al.* Wintertime organic and inorganic aerosols in Lanzhou, China: sources, processes, and comparison with the results during summer. *Atmos. Chem. Phys.* **16**, 14937-14957, doi:10.5194/acp-16-14937-2016 (2016).
91 Huang, R.-J. *et al.* Primary emissions versus secondary formation of fine particulate matter in the most polluted city (Shijiazhuang) in North China. *Atmos. Chem. Phys.* **19**, 2283-2298, doi:10.5194/acp-19-2283-2019 (2019).
92 Wang, Y. *et al.* Chemical composition, sources and secondary processes of aerosols in Baoji city of northwest China. *Atmos. Environ.* **158**, 128-137, doi:10.1016/j.atmosenv.2017.03.026 (2017).

93 Xu, W. *et al.* Effects of Aqueous-Phase and Photochemical Processing on Secondary Organic Aerosol Formation and Evolution in Beijing, China. *Environ. Sci. Technol.* **51**, 762-770, doi:10.1021/acs.est.6b04498 (2017).

94 Wang, Q. *et al.* Characterization of submicron aerosols at a suburban site in central China. *Atmos. Environ.* **131**, 115-123, doi:10.1016/j.atmosenv.2016.01.054 (2016).

95 Hua, Y. *et al.* Characteristics and sources of aerosol pollution at a polluted rural site southwest in Beijing, China. *Sci. Total Environ.* **626**, 519-527, doi:10.1016/j.scitotenv.2018.01.047 (2018).

96 Zhang, X. *et al.* Chemical characterization of submicron aerosol particles during wintertime in a northwest city of China using an Aerodyne aerosol mass spectrometry. *Environ. Pollut.* **222**, 567-582, doi:10.1016/j.envpol.2016.11.012 (2017).

97 Qin, Y. M. *et al.* Impacts of traffic emissions on atmospheric particulate nitrate and organics at a downwind site on the periphery of Guangzhou, China. *Atmos. Chem. Phys.* **17**, 10245-10258, doi:10.5194/acp-17-10245-2017 (2017).

98 Xu, W. *et al.* Changes in Aerosol Chemistry From 2014 to 2016 in Winter in Beijing: Insights From High-Resolution Aerosol Mass Spectrometry. *J. Geophys. Res. Atmos.* **124**, 1132-1147, doi:10.1029/2018JD029245 (2019).

99 Zhou, W. *et al.* Vertical Characterization of Aerosol Particle Composition in Beijing, China: Insights From 3-Month Measurements With Two Aerosol Mass Spectrometers. *J. Geophys. Res. Atmos.* **123**, 13016-13029, doi:10.1029/2018jd029337 (2018).

100 Li, H. *et al.* Rapid transition in winter aerosol composition in Beijing from 2014 to 2017: response to clean air actions. *Atmos. Chem. Phys.* **19**, 11485-11499, doi:10.5194/acp-19-11485-2019 (2019).

101 Cao, L.-M., Huang, X.-F., Li, Y.-Y., Hu, M. & He, L.-Y. Volatility measurement of atmospheric submicron aerosols in an urban atmosphere in southern China. *Atmos. Chem. Phys.* **18**, 1729-1743, doi:10.5194/acp-18-1729-2018 (2018).

102 Zhang, Y. *et al.* Response of aerosol composition to different emission scenarios in Beijing, China. *Sci. Total Environ.* **571**, 902-908, doi:10.1016/j.scitotenv.2016.07.073 (2016).

103 Zhu, Q. *et al.* Atmospheric aerosol compositions and sources at two national background sites in northern and southern China. *Atmos. Chem. Phys.* **16**, 10283-10297, doi:10.5194/acp-16-10283-2016 (2016).

104 Gu, Y. *et al.* Chemical nature and sources of fine particles in urban Beijing: Seasonality and formation mechanisms. *Environ. Int.* **140**, 105732, doi:10.1016/j.envint.2020.105732 (2020).

105 Zheng, J. *et al.* Influence of biomass burning from South Asia at a high-altitude mountain receptor site in China. *Atmos. Chem. Phys.* **17**, 6853-6864, doi:10.5194/acp-17-6853-2017 (2017).

106 Wang, J. *et al.* Highly time-resolved urban aerosol characteristics during springtime in Yangtze River Delta, China: insights from soot particle aerosol mass spectrometry. *Atmos. Chem. Phys.* **16**, 9109-9127, doi:10.5194/acp-16-9109-2016 (2016).

107 Cao, L. *et al.* Chemical characterization and source apportionment of atmospheric submicron particles on the western coast of Taiwan Strait, China. *J. Environ. Sci.* **52**, 293-304, doi:10.1016/j.jes.2016.09.018 (2017).

108 Wang, J. *et al.* First Chemical Characterization of Refractory Black Carbon Aerosols and Associated Coatings over the Tibetan Plateau (4730 m a.s.l). *Environ. Sci. Technol.* **51**, 14072-14082, doi:10.1021/acs.est.7b03973 (2017).

109 Duan, J. *et al.* Summertime and wintertime atmospheric processes of secondary aerosol in Beijing. *Atmos. Chem. Phys.* **20**, 3793-3807, doi:10.5194/acp-20-3793-2020 (2020).
110 Zhao, Q. *et al.* Chemical characterization and source identification of submicron aerosols from a year-long real-time observation at a rural site of Shanghai using an Aerosol Chemical Speciation Monitor. *Atmos. Res.* **246**, 105154, doi:10.1016/j.atmosres.2020.105154 (2020).
111 Duan, J. *et al.* Distinctions in source regions and formation mechanisms of secondary aerosol in Beijing from summer to winter. *Atmos. Chem. Phys.* **19**, 10319-10334, doi:10.5194/acp-19-10319-2019 (2019).
112 Zhao, J. *et al.* Insights into aerosol chemistry during the 2015 China Victory Day parade: results from simultaneous measurements at ground level and 260 m in Beijing. *Atmos. Chem. Phys.* **17**, 3215-3232, doi:10.5194/acp-17-3215-2017 (2017).
113 Zhang, Y. *et al.* Field characterization of the $PM_{2.5}$ Aerosol Chemical Speciation Monitor: insights into the composition, sources, and processes of fine particles in eastern China. *Atmos. Chem. Phys.* **17**, 14501-14517, doi:10.5194/acp-17-14501-2017 (2017).
114 Xu, P. *et al.* Characterization and source identification of submicron aerosol during serious haze pollution periods in Beijing. *J. Environ. Sci.* **112**, 25-37, doi:10.1016/j.jes.2021.04.005 (2022).
115 Li, H. *et al.* Wintertime aerosol chemistry and haze evolution in an extremely polluted city of the North China Plain: significant contribution from coal and biomass combustion. *Atmos. Chem. Phys.* **17**, 4751-4768, doi:10.5194/acp-17-4751-2017 (2017).
116 Cao, L. M., Huang, X. F., Wang, C., Zhu, Q. & He, L. Y. Characterization of submicron aerosol volatility in the regional atmosphere in Southern China. *Chemosphere* **236**, 124383, doi:10.1016/j.chemosphere.2019.124383 (2019).
117 Zhang, X., Xu, J., Kang, S., Liu, Y. & Zhang, Q. Chemical characterization of long-range transport biomass burning emissions to the Himalayas: insights from high-resolution aerosol mass spectrometry. *Atmos. Chem. Phys.* **18**, 4617-4638, doi:10.5194/acp-18-4617-2018 (2018).
118 Zhang, Y. *et al.* Aerosol chemistry and particle growth events at an urban downwind site in North China Plain. *Atmos. Chem. Phys.* **18**, 14637-14651, doi:10.5194/acp-18-14637-2018 (2018).
119 Li, K. *et al.* Chemical characteristics and sources of PM_1 during the 2016 summer in Hangzhou. *Environ. Pollut.* **232**, 42-54, doi:10.1016/j.envpol.2017.09.016 (2018).
120 Zhu, W. *et al.* Seasonal variation of aerosol compositions in Shanghai, China: Insights from particle aerosol mass spectrometer observations. *Sci. Total Environ.* **771**, 144948, doi:10.1016/j.scitotenv.2021.144948 (2021).
121 Zheng, Y. *et al.* Secondary Formation of Submicron and Supermicron Organic and Inorganic Aerosols in a Highly Polluted Urban Area. *J. Geophys. Res. Atmos.* **128**, e2022JD037865, doi:10.1029/2022jd037865 (2023).
122 Zhang, Y. *et al.* On the fossil and non-fossil fuel sources of carbonaceous aerosol with radiocarbon and AMS-PMF methods during winter hazy days in a rural area of North China plain. *Environ. Res.* **208**, 112672, doi:10.1016/j.envres.2021.112672 (2022).
123 Zhao, J. *et al.* Organic Aerosol Processing During Winter Severe Haze Episodes in Beijing. *J. Geophys. Res. Atmos.* **124**, 10248-10263, doi:10.1029/2019jd030832 (2019).
124 Li, H. *et al.* Nitrate-driven urban haze pollution during summertime over the North China Plain. *Atmos. Chem. Phys.* **18**, 5293-5306, doi:10.5194/acp-18-5293-2018 (2018).
125 Zhang, X., Xu, J., Kang, S., Zhang, Q. & Sun, J. Chemical characterization and sources of submicron aerosols in the northeastern Qinghai–Tibet Plateau: insights from high-

resolution mass spectrometry. *Atmos. Chem. Phys.* **19**, 7897-7911, doi:10.5194/acp-19-7897-2019 (2019).

126 Zhang, Y. *et al.* Chemical composition and sources of submicron aerosol in a coastal city of China: Results from the 2017 BRICS summit study. *Sci. Total Environ.* **741**, 140470, doi:10.1016/j.scitotenv.2020.140470 (2020).

127 Zhou, W. *et al.* Response of aerosol chemistry to clean air action in Beijing, China: Insights from two-year ACSM measurements and model simulations. *Environ. Pollut.* **255**, 113345, doi:10.1016/j.envpol.2019.113345 (2019).

128 Wang, Y. *et al.* Aerosol composition, sources, and secondary processing during autumn at a regional site in the Beijing–Tianjin–Hebei region. *Particuology* **75**, 177-184, doi:10.1016/j.partic.2022.07.011 (2023).

129 Xiao, Y. *et al.* Insights into aqueous-phase and photochemical formation of secondary organic aerosol in the winter of Beijing. *Atmos. Environ.* **259**, 118535, doi:10.1016/j.atmosenv.2021.118535 (2021).

130 Yao, D. *et al.* Characteristics, sources and evolution processes of atmospheric organic aerosols at a roadside site in Hong Kong. *Atmos. Environ.* **252**, 118298, doi:10.1016/j.atmosenv.2021.118298 (2021).

131 Wang, Y. *et al.* Chemical composition and sources of submicron aerosols in winter at a regional site in Beijing-Tianjin-Hebei region: Implications for the Joint Action Plan. *Sci. Total Environ.* **719**, 137547, doi:10.1016/j.scitotenv.2020.137547 (2020).

132 Guo, J. *et al.* Characterization of submicron particles by time-of-flight aerosol chemical speciation monitor (ToF-ACSM) during wintertime: aerosol composition, sources, and chemical processes in Guangzhou, China. *Atmos. Chem. Phys.* **20**, 7595-7615, doi:10.5194/acp-20-7595-2020 (2020).

133 Chen, Y. P. *et al.* Chemical composition of NR-PM_1 in a coastal city of Southeast China: Temporal variations and formation pathways. *Atmos. Environ.* **285**, 119243, doi:10.1016/j.atmosenv.2022.119243 (2022).

134 Zheng, Y. *et al.* Characterization of anthropogenic organic aerosols by TOF-ACSM with the new capture vaporizer. *Atmos. Meas. Tech.* **13**, 2457-2472, doi:10.5194/amt-13-2457-2020 (2020).

135 Liu, T. *et al.* Secondary Organic Aerosol Formation from Urban Roadside Air in Hong Kong. *Environ. Sci. Technol.* **53**, 3001-3009, doi:10.1021/acs.est.8b06587 (2019).

136 Huang, X. *et al.* Real-time non-refractory PM_1 chemical composition, size distribution and source apportionment at a coastal industrial park in the Yangtze River Delta region, China. *Sci. Total Environ.* **763**, 142968, doi:10.1016/j.scitotenv.2020.142968 (2021).

137 Zhong, H. *et al.* Measurement report: On the contribution of long-distance transport to the secondary aerosol formation and aging. *Atmos. Chem. Phys.* **22**, 9513-9524, doi:10.5194/acp-22-9513-2022 (2022).

138 Xu, W. *et al.* Summertime aerosol volatility measurements in Beijing, China. *Atmos. Chem. Phys.* **19**, 12, doi:10.5194/acp-19-10205-2019 (2019).

139 Chen, T. *et al.* Chemical characterization of submicron aerosol in summertime Beijing: A case study in southern suburbs in 2018. *Chemosphere* **247**, 125918, doi:10.1016/j.chemosphere.2020.125918 (2020).

140 Lei, L. *et al.* Fine particle characterization in a coastal city in China: composition, sources, and impacts of industrial emissions. *Atmos. Chem. Phys.* **20**, 2877-2890, doi:10.5194/acp-20-2877-2020 (2020).

141 Chen, W. *et al.* Real-Time Characterization of Aerosol Compositions, Sources, and Aging Processes in Guangzhou During PRIDE-GBA 2018 Campaign. *J. Geophys. Res. Atmos.* **126**, e2021JD035114, doi:10.1029/2021JD035114 (2021).
142 Li, Z. J. *et al.* Nitrate and secondary organic aerosol dominated particle light extinction in Beijing due to clean air action. *Atmos. Environ.* **269**, 118833, doi:10.1016/j.atmosenv.2021.118833 (2022).
143 Cui, S. *et al.* Chemical properties, sources and size-resolved hygroscopicity of submicron black-carbon-containing aerosols in urban Shanghai. *Atmos. Chem. Phys.* **22**, 8073-8096, doi:10.5194/acp-22-8073-2022 (2022).
144 Yao, D., Guo, H., Lyu, X., Lu, H. & Huo, Y. Secondary organic aerosol formation at an urban background site on the coastline of South China: Precursors and aging processes. *Environ. Pollut.* **309**, 119778, doi:10.1016/j.envpol.2022.119778 (2022).
145 Li, J. *et al.* Highly time-resolved chemical characterization and implications of regional transport for submicron aerosols in the North China Plain. *Sci. Total Environ.* **705**, 135803, doi:10.1016/j.scitotenv.2019.135803 (2020).
146 Xu, W. *et al.* Organic aerosol volatility and viscosity in the North China Plain: contrast between summer and winter. *Atmos. Chem. Phys.* **21**, 5463-5476, doi:10.5194/acp-21-5463-2021 (2021).
147 Chen, C. *et al.* Aerosol water content enhancement leads to changes in the major formation mechanisms of nitrate and secondary organic aerosols in winter over the North China Plain. *Environ. Pollut.* **287**, 117625, doi:10.1016/j.envpol.2021.117625 (2021).
148 Tang, C. *et al.* Chemical characteristics and regional transport of submicron particulate matter at a suburban site near Lanzhou, China. *Environ. Res.* **212**, 113179, doi:10.1016/j.envres.2022.113179 (2022).
149 Sun, Y. *et al.* Chemical Differences Between PM_1 and $PM_{2.5}$ in Highly Polluted Environment and Implications in Air Pollution Studies. *Geophys. Res. Lett.* **47**, e2019GL086288, doi:10.1029/2019GL086288 (2020).
150 Cao, L.-M. *et al.* Aqueous aging of secondary organic aerosol coating onto black carbon: Insights from simultaneous L-ToF-AMS and SP-AMS measurements at an urban site in southern China. *J. Clean. Prod.* **330**, 129888, doi:10.1016/j.jclepro.2021.129888 (2022).
151 Feng, Z. *et al.* Highly oxidized organic aerosols in Beijing: Possible contribution of aqueous-phase chemistry. *Atmos. Environ.* **273**, 118971, doi: 10.1016/j.atmosenv.2022.118971 (2022).
152 Duan, J. *et al.* Measurement report: Large contribution of biomass burning and aqueous-phase processes to the wintertime secondary organic aerosol formation in Xi'an, Northwest China. *Atmos. Chem. Phys.* **22**, 10139-10153, doi:10.5194/acp-22-10139-2022 (2022).
153 Wang, Q. *et al.* Seasonal characterization of aerosol composition and sources in a polluted city in Central China. *Chemosphere* **258**, 127310, doi:10.1016/j.chemosphere.2020.127310 (2020).
154 Hu, R. *et al.* Variations and Sources of Organic Aerosol in Winter Beijing under Markedly Reduced Anthropogenic Activities During COVID-2019. *Environ. Sci. Technol.* **56**, 6956-6967, doi:10.1021/acs.est.1c05125 (2022).
155 Wang, Y. *et al.* Response of aerosol composition to the clean air actions in Baoji city of Fen-Wei River Basin. *Environ. Res.* **210**, 112936, doi: 10.1016/j.envres.2022.112936 (2022).

156 Li, J. *et al.* Seasonal variations in the highly time-resolved aerosol composition, sources and chemical processes of background submicron particles in the North China Plain. *Atmos. Chem. Phys.* **21**, 4521-4539, doi:10.5194/acp-21-4521-2021 (2021).

157 Duan, J. *et al.* The formation and evolution of secondary organic aerosol during summer in Xi'an: Aqueous phase processing in fog-rain days. *Sci. Total Environ.* **756**, 144077, doi:10.1016/j.scitotenv.2020.144077 (2021).

158 Chen, T. *et al.* Measurement report: Effects of photochemical aging on the formation and evolution of summertime secondary aerosol in Beijing. *Atmos. Chem. Phys.* **21**, 1341-1356, doi:10.5194/acp-21-1341-2021 (2021).

159 Gu, Y. *et al.* Multiple pathways for the formation of secondary organic aerosol in the North China Plain in summer. *Atmos. Chem. Phys.* **23**, 5419-5433, doi:10.5194/acp-23-5419-2023 (2023).

160 Zhang, X. *et al.* High-resolution physicochemical dataset of atmospheric aerosols over the Tibetan Plateau and its surroundings. *Earth Syst. Sci. Data Discuss.* **2022**, 1-46, doi:10.5194/essd-2022-211 (2022).

161 Li, Z. *et al.* Aerosol characterization in a city in central China plain and implications for emission control. *J Environ Sci* **104**, 242-252, doi:10.1016/j.jes.2020.11.015 (2021).

162 Huang, D. D. *et al.* Comparative Assessment of Cooking Emission Contributions to Urban Organic Aerosol Using Online Molecular Tracers and Aerosol Mass Spectrometry Measurements. *Environ. Sci. Technol.* **55**, 14526-14535, doi:10.1021/acs.est.1c03280 (2021).

163 Zhong, H. *et al.* Enhanced formation of secondary organic aerosol from photochemical oxidation during the COVID-19 lockdown in a background site in Northwest China. *Sci. Total Environ.* **778**, 144947, doi:10.1016/j.scitotenv.2021.144947 (2021).

164 Zhou, W. *et al.* Unexpected Increases of Severe Haze Pollution During the Post COVID-19 Period: Effects of Emissions, Meteorology, and Secondary Production. *J. Geophys. Res. Atmos.* **127**, e2021JD035710, doi:10.1029/2021JD035710 (2022).

165 Xu, J. *et al.* COVID-19 Impact on the Concentration and Composition of Submicron Particulate Matter in a Typical City of Northwest China. *Geophys. Res. Lett.* **47**, e2020GL089035, doi:10.1029/2020GL089035 (2020).

166 Tang, M.-X. *et al.* Decisive role of ozone formation control in winter PM2.5 mitigation in Shenzhen, China. *Environ. Pollut.* **301**, 119027, doi: 10.1016/j.envpol.2022.119027 (2022).

167 Xu, W. *et al.* Secondary organic aerosol formation and aging from ambient air in an oxidation flow reactor during wintertime in Beijing, China. *Environ. Res.* **209**, 112751, doi:10.1016/j.envres.2022.112751 (2022).

168 Hu, W. *et al.* Characterization of submicron aerosols influenced by biomass burning at a site in the Sichuan Basin, southwestern China. *Atmos. Chem. Phys.* **16**, 13213-13230, doi:10.5194/acp-16-13213-2016 (2016).

169 Zhang, Y. M. *et al.* Chemical composition and mass size distribution of PM_1 at an elevated site in central east China. *Atmos. Chem. Phys.* **14**, 12237-12249, doi:10.5194/acp-14-12237-2014 (2014).

170 Sun, Y. *et al.* Aerosol characterization over the North China Plain: Haze life cycle and biomass burning impacts in summer. *J. Geophys. Res. Atmos.* **121**, 2508-2521, doi:10.1002/2015JD024261 (2016).

171 Sun, Y. *et al.* Primary and secondary aerosols in Beijing in winter: sources, variations and processes. *Atmos. Chem. Phys.* **16**, 8309-8329, doi:10.5194/acp-16-8309-2016 (2016).

172 Zhang, J. K. *et al.* New characteristics of submicron aerosols and factor analysis of combined organic and inorganic aerosol mass spectra during winter in Beijing. *Atmos. Chem. Phys. Discuss.* **15**, 18537-18576, doi:10.5194/acpd-15-18537-2015 (2015).
173 Wang, J. *et al.* Influence of regional emission controls on the chemical composition, sources, and size distributions of submicron aerosols: Insights from the 2014 Nanjing Youth Olympic Games. *Sci. Total Environ.* **807**, 150869, doi:10.1016/j.scitotenv.2021.150869 (2022).
174 Zhang, J. K. *et al.* Characterization of submicron particles during biomass burning and coal combustion periods in Beijing, China. *Sci. Total Environ.* **562**, 812-821, doi:10.1016/j.scitotenv.2016.04.015 (2016).
175 Xu, W. *et al.* Aerosol composition, oxidation properties, and sources in Beijing: results from the 2014 Asia-Pacific Economic Cooperation summit study. *Atmos. Chem. Phys.* **15**, 13681-13698, doi:10.5194/acp-15-13681-2015 (2015).
176 Zhang, J. K., Wang, L. L., Wang, Y. H. & Wang, Y. S. Submicron aerosols during the Beijing Asia–Pacific Economic Cooperation conference in 2014. *Atmos. Environ.* **124**, 224-231, doi:10.1016/j.atmosenv.2015.06.049 (2016).
177 Huang, R.-J. *et al.* Contrasting sources and processes of particulate species in haze days with low and high relative humidity in wintertime Beijing. *Atmos. Chem. Phys.* **20**, 9101-9114, doi:10.5194/acp-20-9101-2020 (2020).
178 Xu, J. *et al.* Chemical characteristics of submicron particles at the central Tibetan Plateau: insights from aerosol mass spectrometry. *Atmos. Chem. Phys.* **18**, 427-443, doi:10.5194/acp-18-427-2018 (2018).
179 Fan, X. *et al.* Contrasting size-resolved hygroscopicity of fine particles derived by HTDMA and HR-ToF-AMS measurements between summer and winter in Beijing: the impacts of aerosol aging and local emissions. *Atmos. Chem. Phys.* **20**, 915-929, doi:10.5194/acp-20-915-2020 (2020).
180 Tong, Y. *et al.* Quantification of solid fuel combustion and aqueous chemistry contributions to secondary organic aerosol during wintertime haze events in Beijing. *Atmos. Chem. Phys.* **21**, 9859-9886, doi:10.5194/acp-21-9859-2021 (2021).
181 Cai, J. *et al.* Size-segregated particle number and mass concentrations from different emission sources in urban Beijing. *Atmos. Chem. Phys.* **20**, 12721-12740, doi:10.5194/acp-20-12721-2020 (2020).
182 Yan, Y. C. *et al.* Physiochemistry characteristics and sources of submicron aerosols at the background area of North China Plain: Implication of air pollution control in heating season. *Atmos. Res.* **249**, 105291, doi:10.1016/j.atmosres.2020.105291 (2021).
183 Zeng, L. *et al.* The interplays among meteorology, source, and chemistry in high particulate matter pollution episodes in urban Shanghai, China. *Sci. Total Environ.* **853**, 158347, doi: 10.1016/j.scitotenv.2022.158347 (2022).
184 Li, Y. *et al.* Investigation of sources and formation mechanisms of fine particles and organic aerosols in cold season in Fenhe Plain, China. *Atmos. Res.* **268**, 106018, doi:10.1016/j.atmosres.2022.106018 (2022).
185 Li, Y. J. *et al.* Real-time chemical characterization of atmospheric particulate matter in China: A review. *Atmos. Environ.* **158**, 270-304, doi:10.1016/j.atmosenv.2017.02.027 (2017).
186 Li, Y. *et al.* Characteristics of atmospheric intermediate volatility organic compounds (IVOCs) in winter and summer under different air pollution levels. *Atmos. Environ.* **210**, 58-65, doi:10.1016/j.atmosenv.2019.04.041 (2019).

187 Wang, P. *et al.* Concentration, composition and variation of ambient IVOCs in Shanghai Port during the G20 summit. *Geochimica*, 313-321, doi:10.19700/j.0379-1726.2018.03.008 (2018).
188 Ren, B. *et al.* An alternative semi-quantitative GC/MS method to estimate levels of airborne intermediate volatile organic compounds (IVOCs) in ambient air. *Atmos Environ-X* **6**, 100075, doi:10.1016/j.aeaoa.2020.100075 (2020).
189 Lu, J. *et al.* An online method for monitoring atmospheric intermediate volatile organic compounds with a thermal desorption-gas chromatography/mass spectrometry. *J. Chromatogr. A* **1677**, 463299, doi:10.1016/j.chroma.2022.463299 (2022).
190 Fang, H. *et al.* Intermediate-Volatility Organic Compounds Observed in a Coastal Megacity: Importance of Non-Road Source Emissions. *J. Geophys. Res. Atmos.* **127**, e2022JD037301, doi:10.1029/2022JD037301 (2022).
191 Zhao, Y. *et al.* Intermediate-volatility organic compounds: a large source of secondary organic aerosol. *Environ. Sci. Technol.* **48**, 13743-13750, doi:10.1021/es5035188 (2014).
192 Karnezi, E., Riipinen, I. & Pandis, S. N. Measuring the atmospheric organic aerosol volatility distribution: a theoretical analysis. *Atmos. Meas. Tech.* **7**, 2953-2965, doi:10.5194/amt-7-2953-2014 (2014).
193 Feng, T. *et al.* Impact of aging on the sources, volatility, and viscosity of organic aerosols in Chinese outflows. *Atmos. Chem. Phys.* **23**, 611-636, doi:10.5194/acp-23-611-2023 (2023).
194 Faulhaber, A. E. *et al.* Characterization of a thermodenuder-particle beam mass spectrometer system for the study of organic aerosol volatility and composition. *Atmos. Meas. Tech.* **2**, 15-31, doi:10.5194/amt-2-15-2009 (2009).
195 Tan, Z. *et al.* Radical chemistry at a rural site (Wangdu) in the North China Plain: observation and model calculations of OH, HO_2 and RO_2 radicals. *Atmos. Chem. Phys.* **17**, 663-690, doi:10.5194/acp-17-663-2017 (2017).
196 Tan, Z. *et al.* Wintertime photochemistry in Beijing: observations of RO_x radical concentrations in the North China Plain during the BEST-ONE campaign. *Atmos. Chem. Phys.* **18**, 12391-12411, doi:10.5194/acp-18-12391-2018 (2018).
197 Ma, X. *et al.* Winter photochemistry in Beijing: Observation and model simulation of OH and HO_2 radicals at an urban site. *Sci. Total Environ.* **685**, 85-95, doi:10.1016/j.scitotenv.2019.05.329 (2019).
198 Tan, Z. *et al.* Experimental budgets of OH, HO_2, and RO_2 radicals and implications for ozone formation in the Pearl River Delta in China 2014. *Atmos. Chem. Phys.* **19**, 7129-7150, doi:10.5194/acp-19-7129-2019 (2019).
199 Slater, E. J. *et al.* Elevated levels of OH observed in haze events during wintertime in central Beijing. *Atmos. Chem. Phys.* **20**, 14847-14871, doi:10.5194/acp-20-14847-2020 (2020).
200 Whalley, L. K. *et al.* Evaluating the sensitivity of radical chemistry and ozone formation to ambient VOCs and NO_x in Beijing. *Atmos. Chem. Phys.* **21**, 2125-2147, doi:10.5194/acp-21-2125-2021 (2021).
201 Yang, X. *et al.* Observations and modeling of OH and HO_2 radicals in Chengdu, China in summer 2019. *Sci. Total Environ.* **772**, 144829, doi:10.1016/j.scitotenv.2020.144829 (2021).
202 Ma, X. *et al.* OH and HO_2 radical chemistry at a suburban site during the EXPLORE-YRD campaign in 2018. *Atmos. Chem. Phys.* **22**, 7005-7028, doi:10.5194/acp-22-7005-2022 (2022).

203 Yang, X. *et al.* Radical chemistry in the Pearl River Delta: observations and modeling of OH and HO_2 radicals in Shenzhen in 2018. *Atmos. Chem. Phys.* **22**, 12525-12542, doi:10.5194/acp-22-12525-2022 (2022).

204 Zhai, T. *et al.* Elucidate the formation mechanism of particulate nitrate based on direct radical observations in the Yangtze River Delta summer 2019. *Atmos. Chem. Phys.* **23**, 2379-2391, doi:10.5194/acp-23-2379-2023 (2023).

205 Liu, Y. *et al.* A Comprehensive Model Test of the HONO Sources Constrained to Field Measurements at Rural North China Plain. *Environ. Sci. Technol.* **53**, 3517-3525, doi:10.1021/acs.est.8b06367 (2019).

206 Liu, Y. *et al.* Semi-quantitative understanding of source contribution to nitrous acid (HONO) based on 1 year of continuous observation at the SORPES station in eastern China. *Atmos. Chem. Phys.* **19**, 13289-13308, doi:10.5194/acp-19-13289-2019 (2019).

207 Gu, R. *et al.* Atmospheric nitrous acid (HONO) at a rural coastal site in North China: Seasonal variations and effects of biomass burning. *Atmos. Environ.* **229**, 117429, doi:10.1016/j.atmosenv.2020.117429 (2020).

208 Hao, Q., Jiang, N., Zhang, R., Yang, L. & Li, S. Characteristics, sources, and reactions of nitrous acid during winter at an urban site in the Central Plains Economic Region in China. *Atmos. Chem. Phys.* **20**, 7087-7102, doi:10.5194/acp-20-7087-2020 (2020).

209 Jia, C. *et al.* Pollution characteristics and potential sources of nitrous acid (HONO) in early autumn 2018 of Beijing. *Sci. Total Environ.* **735**, 139317, doi:10.1016/j.scitotenv.2020.139317 (2020).

210 Liu, Y. *et al.* The promotion effect of nitrous acid on aerosol formation in wintertime in Beijing: the possible contribution of traffic-related emissions. *Atmos. Chem. Phys.* **20**, 13023-13040, doi:10.5194/acp-20-13023-2020 (2020).

211 Meng, F. *et al.* High-resolution vertical distribution and sources of HONO and NO_2 in the nocturnal boundary layer in urban Beijing, China. *Atmos. Chem. Phys.* **20**, 5071-5092, doi:10.5194/acp-20-5071-2020 (2020).

212 Shi, X. *et al.* Budget of nitrous acid and its impacts on atmospheric oxidative capacity at an urban site in the central Yangtze River Delta region of China. *Atmos. Environ.* **238**, 117725, doi:10.1016/j.atmosenv.2020.117725 (2020).

213 Xue, C. *et al.* HONO Budget and Its Role in Nitrate Formation in the Rural North China Plain. *Environ. Sci. Technol.* **54**, 11048-11057, doi:10.1021/acs.est.0c01832 (2020).

214 Zheng, J. *et al.* Contribution of nitrous acid to the atmospheric oxidation capacity in an industrial zone in the Yangtze River Delta region of China. *Atmos. Chem. Phys.* **20**, 5457-5475, doi:10.5194/acp-20-5457-2020 (2020).

215 Ge, Y. F. *et al.* Seasonality of nitrous acid near an industry zone in the Yangtze River Delta region of China: Formation mechanisms and contribution to the atmospheric oxidation capacity. *Atmos. Environ.* **254**, 118420, doi:10.1016/j.atmosenv.2021.118420 (2021).

216 Zheng, Y. *et al.* Precursors and Pathways Leading to Enhanced Secondary Organic Aerosol Formation during Severe Haze Episodes. *Environ. Sci. Technol.* **55**, 15680-15693, doi:10.1021/acs.est.1c04255 (2021).

217 Gkatzelis, G. I. *et al.* Uptake of Water-soluble Gas-phase Oxidation Products Drives Organic Particulate Pollution in Beijing. *Geophys. Res. Lett.* **48**, e2020GL091351, doi:10.1029/2020GL091351 (2021).

218 Aiken, A. C. *et al.* O/C and OM/OC Ratios of Primary, Secondary, and Ambient Organic Aerosols with High-Resolution Time-of-Flight Aerosol Mass Spectrometry. *Environ. Sci. Technol.* **42**, 4478-4485, doi:10.1021/es703009q (2008).

219 Lu, Q. *et al.* Simulation of organic aerosol formation during the CalNex study: updated mobile emissions and secondary organic aerosol parameterization for intermediate-volatility organic compounds. *Atmos. Chem. Phys.* **20**, 4313-4332, doi:10.5194/acp-20-4313-2020 (2020).
220 Jo, D. S., Park, R. J., Kim, M. J. & Spracklen, D. V. Effects of chemical aging on global secondary organic aerosol using the volatility basis set approach. *Atmos. Environ.* **81**, 230-244, doi:10.1016/j.atmosenv.2013.08.055 (2013).
221 Brewer, J. F. *et al.* A Scheme for Representing Aromatic Secondary Organic Aerosols in Chemical Transport Models: Application to Source Attribution of Organic Aerosols Over South Korea During the KORUS-AQ Campaign. *J. Geophys. Res. Atmos.* **128**, e2022JD037257, doi:10.1029/2022JD037257 (2023).
222 Nault, B. A. *et al.* Secondary organic aerosol production from local emissions dominates the organic aerosol budget over Seoul, South Korea, during KORUS-AQ. *Atmos. Chem. Phys.* **18**, 17769-17800, doi:10.5194/acp-18-17769-2018 (2018).
223 Hodzic, A. *et al.* Rethinking the global secondary organic aerosol (SOA) budget: stronger production, faster removal, shorter lifetime. *Atmos. Chem. Phys.* **16**, 7917-7941, doi:10.5194/acp-16-7917-2016 (2016).
224 Hu, W. *et al.* Oxidation Flow Reactor Results in a Chinese Megacity Emphasize the Important Contribution of S/IVOCs to Ambient SOA Formation. *Environ. Sci. Technol.*, doi:10.1021/acs.est.1c03155 (2021).
225 Zheng, H. *et al.* Trends of Full-Volatility Organic Emissions in China from 2005 to 2019 and Their Organic Aerosol Formation Potentials. *Environ. Sci. Technol. Let.* **10**, 137-144, doi:10.1021/acs.estlett.2c00944 (2023).
226 Wu, L. *et al.* A gridded emission inventory of semi-volatile and intermediate volatility organic compounds in China. *Sci. Total Environ.* **761**, 143295, doi:10.1016/j.scitotenv.2020.143295 (2021).
227 Zhao, J. *et al.* An updated comprehensive IVOC emission inventory for mobile sources in China. *Sci. Total Environ.* **851**, 158312, doi:10.1016/j.scitotenv.2022.158312 (2022).
228 Wang, A. *et al.* Measurement-based intermediate volatility organic compound emission inventory from on-road vehicle exhaust in China. *Environ Pollut* **310**, 119887, doi:10.1016/j.envpol.2022.119887 (2022).
229 Pye, H. O. T. *et al.* Linking gas, particulate, and toxic endpoints to air emissions in the Community Regional Atmospheric Chemistry Multiphase Mechanism (CRACMM). *Atmos. Chem. Phys.* **23**, 5043-5099, doi:10.5194/acp-23-5043-2023 (2023).
230 Wang, X., Dickinson, R. E., Su, L., Zhou, C. & Wang, K. $PM_{2.5}$ Pollution in China and How It Has Been Exacerbated by Terrain and Meteorological Conditions. *Bull. Am. Meteorol. Soc.* **99**, 105-120, doi:10.1175/Bams-D-16-0301.1 (2018).
231 Zhang, J., Huff Hartz, K. E., Pandis, S. N. & Donahue, N. M. Secondary organic aerosol formation from limonene ozonolysis: homogeneous and heterogeneous influences as a function of NO_x. *J. Phys. Chem. A* **110**, 11053-11063, doi:10.1021/jp062836f (2006).
232 Ng, N. L. *et al.* Effect of NOx level on secondary organic aerosol (SOA) formation from the photooxidation of terpenes. *Atmos. Chem. Phys.* **7**, 5159-5174, doi:10.5194/acp-7-5159-2007 (2007).
233 Eddingsaas, N. C. *et al.* α-pinene photooxidation under controlled chemical conditions – Part 2: SOA yield and composition in low- and high-NO_x environments. *Atmos. Chem. Phys.* **12**, 7413-7427, doi:10.5194/acp-12-7413-2012 (2012).
234 Han, Y., Stroud, C. A., Liggio, J. & Li, S. M. The effect of particle acidity on secondary organic aerosol formation from α-pinene photooxidation under atmospherically

relevant conditions. *Atmos. Chem. Phys.* **16**, 13929-13944, doi:10.5194/acp-16-13929-2016 (2016).

235 Sarrafzadeh, M. *et al.* Impact of NOx and OH on secondary organic aerosol formation from β-pinene photooxidation. *Atmos. Chem. Phys.* **16**, 11237-11248, doi:10.5194/acp-16-11237-2016 (2016).

236 Shilling, J. E. *et al.* Particle mass yield in secondary organic aerosol formed by the dark ozonolysis of α-pinene. *Atmos. Chem. Phys.* **8**, 2073-2088, doi:10.5194/acp-8-2073-2008 (2008).

237 Zhao, D. F. *et al.* Secondary organic aerosol formation from hydroxyl radical oxidation and ozonolysis of monoterpenes. *Atmos. Chem. Phys.* **15**, 991-1012, doi:10.5194/acp-15-991-2015 (2015).

238 Ahlberg, E. *et al.* Secondary organic aerosol from VOC mixtures in an oxidation flow reactor. *Atmos. Environ.* **161**, 210-220, doi: 10.1016/j.atmosenv.2017.05.005 (2017).

239 Tasoglou, A. & Pandis, S. N. Formation and chemical aging of secondary organic aerosol during the *β*-caryophyllene oxidation. *Atmos. Chem. Phys.* **15**, 6035-6046, doi:10.5194/acp-15-6035-2015 (2015).

240 Chen, Q., Li, Y. L., McKinney, K. A., Kuwata, M. & Martin, S. T. Particle mass yield from *β*-caryophyllene ozonolysis. *Atmos. Chem. Phys.* **12**, 3165-3179, doi:10.5194/acp-12-3165-2012 (2012).

241 Kleindienst, T. E., Edney, E. O., Lewandowski, M., Offenberg, J. H. & Jaoui, M. Secondary Organic Carbon and Aerosol Yields from the Irradiations of Isoprene and α-Pinene in the Presence of NOx and SO2. *Environ. Sci. Technol.* **40**, 3807-3812, doi:10.1021/es052446r (2006).

242 Ng, N. L. *et al.* Secondary organic aerosol formation from *m*-xylene, toluene, and benzene. *Atmos. Chem. Phys.* **7**, 3909-3922, doi:10.5194/acp-7-3909-2007 (2007).

243 Sato, K. *et al.* AMS and LC/MS analyses of SOA from the photooxidation of benzene and 1,3,5-trimethylbenzene in the presence of NO_x: effects of chemical structure on SOA aging. *Atmos. Chem. Phys.* **12**, 4667-4682, doi:10.5194/acp-12-4667-2012 (2012).

244 Nakao, S. *et al.* Density and elemental ratios of secondary organic aerosol: Application of a density prediction method. *Atmos. Environ.* **68**, 273-277, doi:10.1016/j.atmosenv.2012.11.006 (2013).

245 Xu, J. L., Griffin, R. J., Liu, Y., Nakao, S. & Cocker, D. R. Simulated impact of NO_x on SOA formation from oxidation of toluene and m-xylene. *Atmos. Environ.* **101**, 217-225, doi:10.1016/j.atmosenv.2014.11.008 (2015).

246 Song, C., Na, K., Warren, B., Malloy, Q. & Cocker, D. R., 3rd. Secondary Organic Aerosol Formation from *m*-Xylene in the Absence of NO_x. *Environ. Sci. Technol.* **41**, 7409-7416, doi:10.1021/es070429r (2007).

247 Li, L., Tang, P., Nakao, S. & Cocker Iii, D. R. Impact of molecular structure on secondary organic aerosol formation from aromatic hydrocarbon photooxidation under low-NOx conditions. *Atmos. Chem. Phys.* **16**, 10793-10808, doi:10.5194/acp-16-10793-2016 (2016).

248 Presto, A. A., Miracolo, M. A., Donahue, N. M. & Robinson, A. L. Secondary Organic Aerosol Formation from High-NO_x Photo-Oxidation of Low Volatility Precursors: *n*-Alkanes. *Environ. Sci. Technol.* **44**, 2029-2034, doi:10.1021/es903712r (2010).

249 Chan, A. W. H. *et al.* Secondary organic aerosol formation from photooxidation of naphthalene and alkylnaphthalenes: implications for oxidation of intermediate volatility organic compounds (IVOCs). *Atmos. Chem. Phys.* **9**, 3049-3060, doi:10.5194/acp-9-3049-2009 (2009).
